# Quinazoline-Derivatives of Imino-1,2,3-Dithiazoles Promote Biofilm Dispersion of *Pseudomonas aeruginosa*

**DOI:** 10.3390/ph18111733

**Published:** 2025-11-14

**Authors:** Mathieu Gonzalez, Anne-Sophie Tareau, Daphnée de Crozals, Corentin Layec, Nathan Broudic, Magalie Barreau, Adrien Forge, Olivier Lesouhaitier, Corinne Fruit, Sylvie Chevalier, Thierry Besson, Ali Tahrioui

**Affiliations:** 1 Univ Rouen Normandie, Université Caen Normandie, Normandie Univ, CBSA UR 4312, F-76000 Rouen, France; mathieu.gonzalez@univ-rouen.fr (M.G.); anne-sophie.tareau@univ-rouen.fr (A.-S.T.); magalie.barreau@univ-rouen.fr (M.B.); adrien.forge@univ-rouen.fr (A.F.); olivier.lesouhaitier@univ-rouen.fr (O.L.); sylvie.chevalier@univ-rouen.fr (S.C.); 2 Plateforme de Sécurité Sanitaire Ébroïcienne (PS^2^E), Univ Rouen Normandie, Normandie Univ, CBSA UR 4312, F-76000 Rouen, France; 3 Univ Rouen Normandie, INSA Rouen Normandie, Univ Caen Normandie, ENSICAEN, CNRS, Institut CARMeN UMR 6064, F-76000 Rouen, France; daphnee.de-crozals@univ-rouen.fr (D.d.C.); corentin.layec@universite-paris-saclay.fr (C.L.); nathan.broudic@u-paris.fr (N.B.); corinne.fruit@univ-rouen.fr (C.F.)

**Keywords:** *Pseudomonas aeruginosa*, *N*-arylimino-1,2,3-dithiazoles, biofilm dispersion

## Abstract

**Background/Objectives:** Biofilm-associated infections pose a major clinical challenge since bacteria within biofilms exhibit highly antibiotic tolerance. *Pseudomonas aeruginosa* forms persistent biofilms that cause chronic infections in vulnerable patients, including those with cystic fibrosis, burns, or medical implants. Such biofilm-associated chronic infections require prolonged treatments that promote antimicrobial resistance. To address this, recent strategies focus on enhancing biofilm dispersion. **Methods:** Thirty-six *N*-arylimino-1,2,3-dithiazoles were screened for their biofilm dispersal activity using a crystal violet assay. Their cytotoxicity was assessed on A549 and HaCat eukaryotic cells. Moreover, their influence on bacterial growth and virulence was investigated. Lastly, fluorescence anisotropy was used to measure membrane fluidity to obtain the first insights on the mechanism of action of these chemicals. **Results:** Our results showed that quinazoline-derivatives of imino-1,2,3-dithiazoles display biofilm dispersion activity. These compounds do not increase virulence through pyocyanin production, do not modify the growth kinetics of *P. aeruginosa,* and do not show cytotoxicity towards eucaryotic cells. **Conclusions:** These findings highlight the potential use of *N*-arylimino-1,2,3-dithiazole-derived compounds as safe and effective dispersal agents of *P. aeruginosa* biofilms.

## 1. Introduction

Bacteria exist as free-living cells corresponding to the planktonic lifestyle or as aggregates, known as biofilms. Bacterial biofilms are communities protected by a self-synthesized matrix composed of polysaccharides, proteins, lipids, and extracellular DNA, collectively known as extracellular polymeric substances [1,2]. These biofilms form when bacteria irreversibly attach to a surface and start cell division. In a medical context, bacterial biofilms may develop on many surfaces, which can be biotic (skin, tissues, mucus) or abiotic (medical devices) [3,4]. Biofilm lifestyle provides bacteria with numerous survival advantages, including physical protection from the host immune system, water retention and tolerance to desiccation, nutrient sorption and storage, adhesion to the infection site, and cell aggregation, leading to the coordination of virulence factors expression via quorum sensing [5,6]. Additionally, the biofilm is responsible for up to a 1000-fold increase in antibiotic tolerance due to the physical impedance and enzymatic inactivation of the drugs, coupled with the lowered metabolic rates of biofilm cells [7,8]. Therefore, biofilms are highly recalcitrant and are associated with chronic, non-healing infections. It is estimated that biofilms are involved in around 60–80% of all infectious diseases, among which more than 90% of all chronic wound infections. Thus, biofilm-associated infections are a major public health problem, since they involve heavy treatments, exposing bacteria to a selection pressure, thus increasing the risks of developing antibiotic resistance [9,10].

**Scheme 1:**
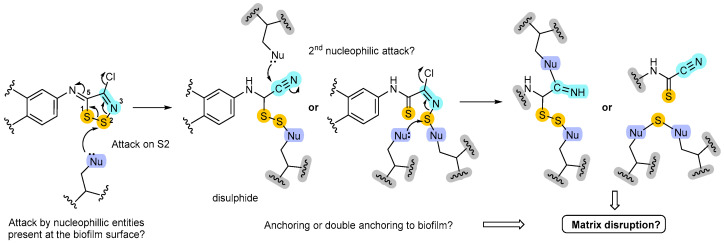
Hypothesis on the possibility of anchoring imino-1,2,3-dithiazoles to the surface of a biofilm.

The opportunistic human pathogen *Pseudomonas aeruginosa* is a Gram-negative bacillus that causes infections in immunocompromised patients. Classified in the ESKAPE pathogens group since 2004 by the Centers for Disease Control and Prevention, it is considered one of the main pathogenic bacteria responsible for hospital-acquired infections. In 2017 and more recently in 2024, *P. aeruginosa* was classified by the World Health Organization as a Critical (2017) or High (2024) Bacterial Priority Pathogen [11,12], due to its numerous antibiotic resistances, its high potential to develop new resistances, and the difficulty of discovering new antibiotics. *P. aeruginosa* secretes many pigments, including pyocyanin, which is one of its numerous virulence factors leading to acute infections. Moreover, this bacterium causes chronic infections, generally associated with its biofilm lifestyle [13]. Notably, *P. aeruginosa* is commonly the leading cause of biofilm-associated infections in patients suffering from cystic fibrosis (CF), acquired immunodeficiency syndrome, burn wounds, or with medical implants [13]. In addition to its strong adaptability and resistance against a wide range of antimicrobials, especially carbapenems, its ability to form biofilms makes it even more difficult to eradicate. Consequently, the therapeutic strategies available to treat *P. aeruginosa* biofilm-associated infections are poor, resulting in high levels of morbidity and mortality. Alternative strategies to replace conventional antibiotic treatment are therefore required. One of these strategies is to promote the switch of bacteria from a biofilm lifestyle to a planktonic state, in which they display a more significant susceptibility to antibiotics [14,15,16]. This transition is possible using molecules that are able to induce biofilm dispersion.

(*Z*)-4-Chloro-*N*-(aryl)-5*H*-1,2,3-dithiazol-5-imine derivatives (also called *N*-arylimino-1,2,3-dithiazoles) remain underexplored in medicinal chemistry, despite demonstrating a broad spectrum of emerging pharmacological activities. To date, imino-1,2,3-dithiazoles have been reported to exhibit antifungal, herbicidal, antiviral, antibacterial, antitumoral, and antifibrotic properties with limited cytotoxicity [17]. The antibacterial potential of these compounds was investigated on an array of Gram-negative and Gram-positive bacteria. These studies demonstrated that the antibacterial activity of the tested imino-1,2,3-dithiazoles is effective only on the Gram-positive bacterial strains assayed. In addition, antibacterial activity appeared to depend solely on the 1,2,3-dithiazole ring and was not affected by the benzenic part of the molecules [18,19]. Since the 1990s, it is well known that imino-1,2,3-dithiazoles can undergo various inter- or intramolecular nucleophilic attacks directly on one of the electrophilic sites (S1, S2, and C5) of the dithiazole ring (Scheme 1). The formation of disulfide intermediates resulting from the attack on S2 is frequently involved in the conversion of the starting imines into various heterocyclic systems, each bearing a carbonitrile function which is latent in the dithiazole structure [17]. Based on these considerations, we hypothesize that imino-1,2,3-dithiazoles can be covalently anchored to the surface of a bacterial biofilm, thereby generating a localized chemical signal capable of perturbing and disrupting the structural integrity of the extracellular matrix. Mechanistically, such anchoring could proceed through one or more nucleophilic attacks targeting either the sulfur atoms of the dithiazole ring or the electrophilic carbon of the nitrile function. Potential nucleophilic partners include amino acid side chains with nucleophilic residues (e.g., cysteine, lysine, or serine) as well as anionic functional groups found in saccharide residues within the extracellular polymeric substance of the biofilm (Scheme 1).

Compared with existing dispersal agents, *N*-arylimino-1,2,3-dithiazoles may present several advantages. For instance, the chemical stability and synthetic versatility of these compounds allow systematic structure-activity relationship (SAR) optimization to improve their efficacy and selectivity, which might favor their interactions with biofilm matrix components or membrane targets. Moreover, their small-molecule nature may facilitate penetration into dense biofilm layers and reduce the likelihood of enzymatic degradation, offering a promising platform for clinical applications where biofilm-associated chronic infection control remains a significant challenge.

The current study aimed to evaluate novel (*Z*)-6-[(4-chloro-5*H*-1,2,3-dithiazol-5-ylidene)amino]quinazolin-4-amine derivatives for their capacity to disperse *P. aeruginosa* biofilm. Thirty-six (*Z*)-4-chloro-*N*-(aryl)-5*H*-1,2,3-dithiazol-5-imine derivatives, grouped into five series according to their aromatic substituents, were screened using a crystal violet assay. The bactericidal effect of the most active derivatives on *P. aeruginosa* was determined, and their cytotoxicity was measured on A549 and HaCat eukaryotic cells. Moreover, their influence on a key virulence factor of *P. aeruginosa* was investigated. Lastly, the first indications of the mechanism of action of these chemicals were investigated by measuring membrane fluidity during exposure to the compounds of interest.

## 2. Results and Discussion

### 2.1. Pseudomonas aeruginosa Biofilm Dispersion by N-Arylimino-1,2,3-Dithiazoles

Bacterial infections are typically treated with bacteriostatic and bactericidal agents such as antibiotics to directly target the pathogen. However, biofilms associated with chronic infections are often recalcitrant to antimicrobials [4,7,20,21]. A promising alternative therapeutic strategy to eradicate these biofilms involves the use of dispersal agents [14,22,23]. It is worth noting that biofilm dispersion is a rapid process that occurs within minutes to a few hours in response to chemical or eukaryotic signals [23,24,25]. Early analysis is critical to accurately capture dispersion effects before dispersed cells reattach or form new biofilms. Short-term measurements also minimize secondary effects such as nutrient depletion, accumulation of toxic by-products, or indirect impacts on cell viability, ensuring that observed biomass reduction reflects true dispersal rather than regrowth.

**Figure 1:**
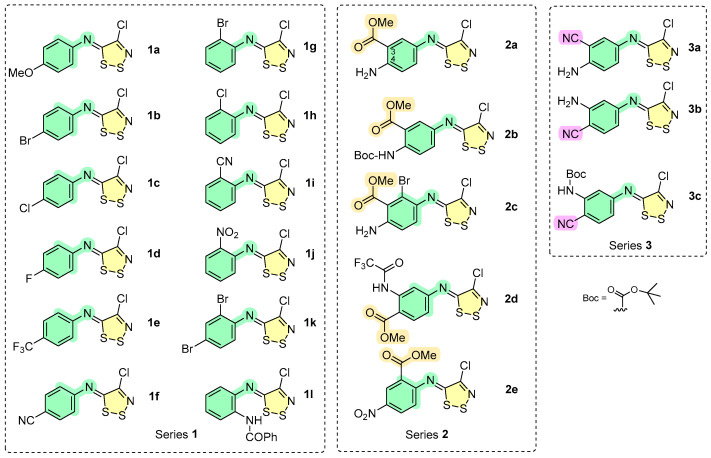
Structures of the first chemical library of *N*-arylimino-1,2,3-dithiazole derivatives tested for their dispersal activity of *P. aeruginosa* biofilm. The series **1a**–**l** consists of monosubstituted derivatives, the series **2a**–**e** comprises polyfunctional compounds derived from anthranilic acid (2-aminobenzoic acid), and the compound series **3a**–**c** is formed from anthranilonitrile (2-aminobenzonitrile).

In the present study, we assessed the potential dispersal effect of *N*-arylimino-1,2,3-dithiazole-derived compounds on 24 h-old established *P. aeruginosa* PA14 biofilms grown in static conditions and exposed for 2 h at 37 °C using a crystal violet staining assay. In the first round of assays, twenty *N*-arylimino-1,2,3-dithiazoles were extracted from our chemical library. These compounds were synthesized in previous studies on functionalized 2-cyanobenzothiazoles [26] and can be divided into three groups (Figure 1). The first group consists of monosubstituted *N*-arylimino-1,2,3-dithiazole derivatives (series **1a**–**l**). The second array comprises polyfunctional compounds derived from anthranilic acid (2-aminobenzoic acid) (series **2a**–**e**) and the last one comprises anthranilonitrile derivatives (2-aminobenzonitriles) (series **3a**–**c**) (Figure 1).

All these *N*-arylimino-1,2,3-dithiazole derivatives were screened for their capacity to disperse *P. aeruginosa* biofilm at four concentrations 10 μM, 1 μM, 0.1 μM, and 0.01 μM (Table 1, for more details see Section 3). Five compounds (**1j** and **1l** from series 1, **2b** from series 2, and **3a** and **3b** from series 3) showed a significant biomass dispersal effect ranging from 27% to 35% at 10 μM. Compounds **1l** and **2b** showed a similar range of dispersal activity at 1 μM, while compound **3a** showed a better biofilm dispersal (45%) at 1 μM (Table 1). However, a weak biofilm dispersal effect (10–14%) was observed at 0.1 μM and 0.01 μM (Table 1).

**Figure 2:**
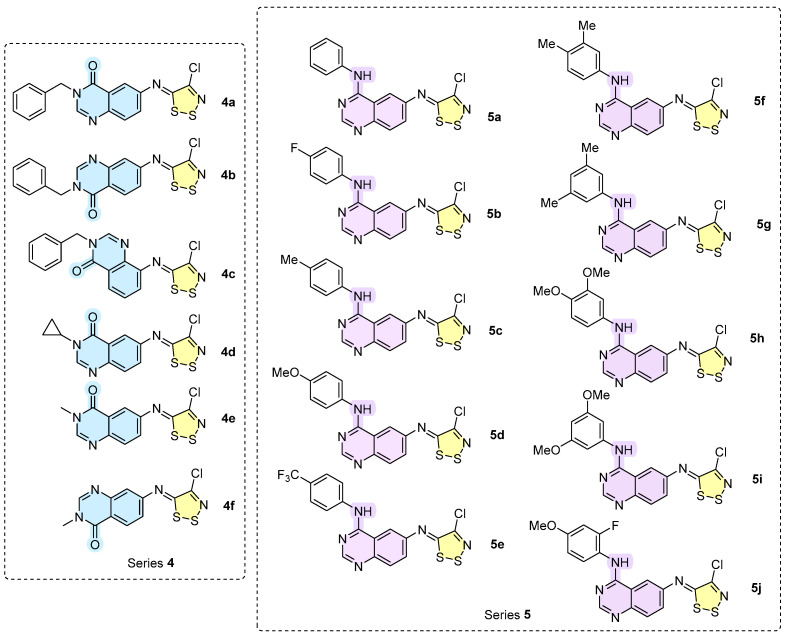
Structures of the second chemical library of *N*-arylimino-1,2,3-dithiazoles of interest. In series **4**, the iminodithiazole ring is associated with a quinazolin-4-one moiety (in blue), while in series **5**, it is a quinazoline system (violet).

Based on these promising results, we speculated that these 1,2,3-dithiazole scaffolds could be used to develop dispersal agents that possess enhanced efficacy for use in the treatment of biofilm-associated chronic infections. It was decided to test a second library of *N*-arylimino-1,2,3-dithiazoles possessing a quinazolin-4-one or quinazoline-type extended aromatic moiety. Of note, recent studies have highlighted the potential pharmacological effects of quinazolinone and quinazoline derivatives, which include anti-cancer, anti-diabetic, anti-malarial, and anti-microbial activities [27,28,29,30,31]. It is also worth mentioning that this second library of *N*-arylimino-1,2,3-dithiazole derivatives was recently used as intermediates in the synthesis of novel thiazole-fused [4,5-*g*] or [5,4-*g*]quinazolines] and their quinazolin-8-one analogs, with potent antiproliferative activity assayed on a panel of cancer cell lines and fibroblasts [32]. Their synthetic route is based on that developed for the preceding series **1–3** (for complete details see Experimental Section). Then, a new set of sixteen *N*-arylimino-1,2,3-dithiazole derivatives (series **4** and **5**) was extracted from our chemical library (Figure 2).

**Figure 3:**
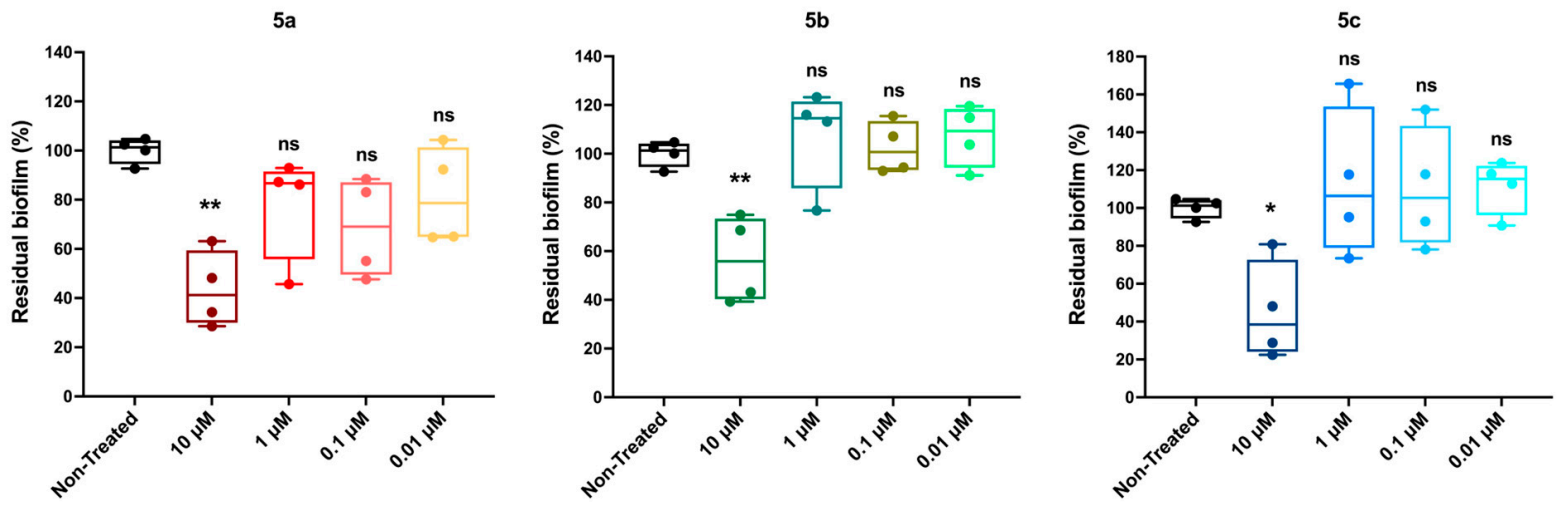
Effect of *N*-arylimino-1,2,3-dithiazole-derived compounds on *P. aeruginosa* biofilm dispersion. The boxplots represent residual biofilm grown in static conditions for 24 h at 37 °C, non-exposed (1% DMSO) or exposed for 2 h to compounds **5a**, **5b**, and **5c** (10 µM, 1 µM, 0.1 µM, 0.01 µM). Bacterial cells within biofilms were stained with crystal violet, and absorbance was read at 595 nm. Data are the results of the analysis of means of twelve technical replicates (control condition), and two technical replicates (for each treated condition) from at least four independent biological experiments (*n* = 4). Statistics were performed by one-way ANOVA followed by Dunnett’s multiple-comparison test. Values that are significantly different are indicated by asterisks as follows: *, *p* < 0.05; **, *p* < 0.01; ns, not significant (*p* > 0.05).

The measurement of the dispersion effect of this second chemical library of *N*-arylimino-1,2,3-dithiazoles derivatives enabled us to identify three significantly active compounds that belong to the fused iminodithiazole and quinazoline moieties class (**5a**, **5b**, and **5c**). Interestingly, the PA14 biofilm dispersion activity of these compounds was about 44–57% upon 2 h exposure and does not appear to be dose-dependent, as a significant dispersal effect was only observed at 10 µM (Table 2, Figure 3).

Since these three compounds were the most efficient, they were selected for further experiments. However, it should be noted that the dispersion activity of **5a** at 1 µM, 0.1 µM, and 0.01 µM is slightly higher than that of the two congeners **5b** and **5c**. To our knowledge, this is the first report demonstrating the potential dispersal activity of *N*-arylimino-1,2,3-dithiazole derivatives on *P. aeruginosa* biofilms.

Previous studies have investigated various classes of agents capable of inducing biofilm dispersion across multiple biofilm-forming pathogens [15,16]. For example, nitric oxide (NO) at low physiological concentrations functions as a biofilm dispersal signal by reducing intracellular c-di-GMP levels in several bacterial genera, including both environmental and clinical isolates [33]. However, when developing NO-based antibiofilm strategies, it is essential to consider the nature of the NO donor, as sodium nitroprusside has been reported to stimulate cell growth and biofilm formation in *P. aeruginosa* [34]. Similarly, biosurfactants such as rhamnolipids can promote biofilm detachment in *P. aeruginosa* [35] by reducing surface tension and altering membrane fluidity. Enzymatic agents, including DNases, dispersin B, and proteases, represent another important class of dispersal agents capable of degrading key components of the biofilm matrix [16,36]; however, these enzymes may lose activity under harsh environmental conditions. In contrast, small heterocyclic compounds such as quinazoline derivatives are more chemically stable and less prone to hydrolysis. Given the complexity of biofilms, complementary investigations are needed to assess the impact of these compounds on biofilm architecture, matrix components (such as polysaccharides, extracellular DNA, proteins) and their toxicity to cells enclosed within biofilms.

### 2.2. Effect of N-Arylimino-1,2,3-Dithiazole Derivatives ***5a***–***c*** on P. aeruginosa Biofilm Formation

**Figure 4:**
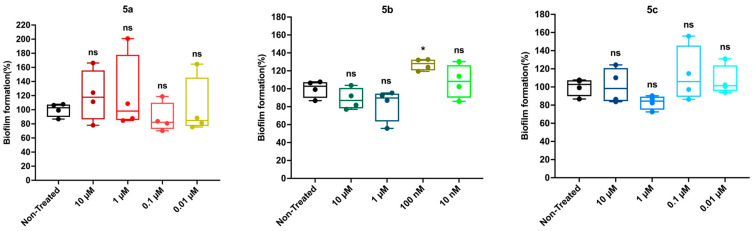
*P. aeruginosa* biofilm formation upon exposure to derivatives of *N*-arylimino-1,2,3-dithiazoles. The boxplots represent biofilms grown in static conditions at 37 °C for 24 h, non-exposed (1% DMSO), or exposed to compounds **5a**, **5b**, and **5c** (10 µM, 1 µM, 0.1 µM, 0.01 µM). Bacterial cells within biofilms were stained with crystal violet, and absorbance was read at 595 nm. Data are the results of the analysis of means of twelve technical replicates (control condition), and two technical replicates (for each treated condition) from at least four independent biological experiments (*n* = 4). Statistics were performed by one-way ANOVA followed by Dunnett’s multiple-comparison test. Values that are significantly different are indicated by asterisks as follows: *, *p* < 0.05; ns, not significant (*p* > 0.05).

Since dispersed bacterial biofilm cells tend to colonize new niches, we next aimed to evaluate the biofilm formation of the *P. aeruginosa* PA14 strain upon exposure to the selected (*Z*)-6-[(4-chloro-5*H*-1,2,3-dithiazol-5-ylidene)amino]-*N*-phenylquinazolin-4-amine derivatives (**5a**, **5b**, and **5c**) at four concentrations (10 μM, 1 μM, 0.1 μM, and 0.01 μM). The compounds were added at the onset of biofilm formation and cultured for 24 h at 37 °C in static conditions. None of the compounds showed a significant inhibition effect on *P. aeruginosa* PA14 biofilm formation, at any of the tested concentrations (Figure 4). These data suggest that the selected *N*-arylimino-1,2,3-dithiazole derivatives **5a**–**c** can act specifically as dispersal agents rather than as inhibitors of *P. aeruginosa* PA14 biofilm formation.

### 2.3. Effect of N-Arylimino-1,2,3-Dithiazoles ***5a***–***c*** on Growth and Virulence of P. aeruginosa

**Figure 5:**
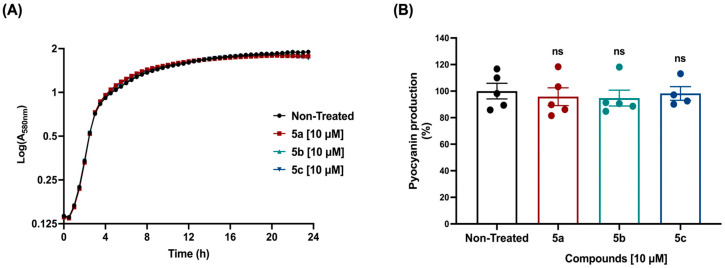
Impact of *N*-arylimino-1,2,3-dithiazoles-derived compounds 5a–c on *P. aeruginosa* growth kinetics and pyocyanin production. (**A**) Aerobic growth (A_580 nm_) in LB medium was monitored at 37 °C during 24 h under shaking conditions, without (non-treated) or with the selected compounds (**5a**, **5b**, and **5c**) at 10 µM. Results are the means of three independent biological experiments (*n* = 3). (**B**) Pyocyanin production (%) by the PA14 strain was measured (A_520 nm_) in the non-treated condition (1% DMSO) or upon 2 h exposure to compounds **5a**, **5b**, and **5c** at 10 µM. Data are the results of the analysis of means of twelve technical replicates (non-treated condition), and two technical replicates (for each treated condition) from at least four independent biological experiments (*n* = 4). Statistics were performed by one-way ANOVA followed by Dunnett’s multiple-comparison test. ns, not significant (*p* > 0.05).

The 1,2,3-dithiazole scaffold has been reported to exhibit significant anti-microbial activity against Gram-positive bacteria, including *Staphylococcus aureus*, *Enterococcus faecalis*, *Streptococcus pyogenes*, *Listeria innocua*, and *Listeria monocytogenes* (MIC of 16 μg/mL) [19,37]. An antibacterial activity of 1,2,3-dithiazole derivatives against the Gram-negative *Escherichia coli* ATCC 11229 was also reported in a patent registered in 1997 by Joseph et al. [38]. In addition, quinazoline derivatives also display antimicrobial activity [39,40]. Since bactericidal activity may exert selective pressure and lead to the emergence of new resistant strains, we sought to find out if the selected quinazoline compounds (**5a**, **5b**, and **5c**) acted solely as dispersal agents or whether they also had a bactericidal effect by assessing their effect on *P. aeruginosa* PA14 strain growth kinetics. We observed that exposure of *P. aeruginosa* to these compounds at 10 µM during 24 h did not modify the growth kinetics as compared to the growth control condition (Figure 5A). This result appears to be in accordance with the studies mentioned above in which no bactericidal activity against *P. aeruginosa* was reported for *N*-arylimino-1,2,3-dithiazole derivatives [18,19,37,38]. The data obtained suggest that the dispersal effect of these three 4-anilinoquinazoline derivatives may not be associated with bactericidal activity.

Since these *N*-arylimino-1,2,3-dithiazole-derived compounds are intended to be used as dispersal agents of *P. aeruginosa* biofilms, they must not increase bacterial virulence. To ascertain this, we assessed the effect of **5a**, **5b**, and **5c** compounds on pyocyanin production, as it is one of the major virulence factors of *P. aeruginosa.* It was found that none of the molecules tested exhibited a significant modification of pyocyanin production at 10 µM as compared to the non-treated condition (Figure 5B). Taken together, these data suggest that the selected imino-1,2,3-dithiazole-derived compounds **5a**, **5b**, and **5c** did not display a bactericidal effect and did not alter the virulence of the *P. aeruginosa* PA14 strain. However, a comprehensive study of the impact of *N*-arylimino-1,2,3-dithiazoles on other virulence factors remains to be carried out.

While biofilm dispersal agents hold potential as adjuvants of antimicrobial therapies for treating biofilm-associated chronic infections, their risks and limitations must be carefully considered [41,42]. Dispersion can release large numbers of planktonic bacteria, potentially leading to acute or systemic infections. Mechanisms of dispersion are complex and species-specific, and incomplete or uncontrolled dispersion may fail to eradicate biofilms or even promote regrowth. The in vivo factors, such as immune responses, nutrient availability, pH, and oxygen levels, can significantly influence outcomes, making laboratory results difficult to translate clinically. Additionally, chemical or enzymatic dispersal inducers may impact beneficial microbiota or host tissues, causing unintended side effects. Overall, the efficacy of biofilm dispersal agents varies considerably depending on the microbial species, strain type, stage of biofilm development, and surrounding environmental conditions [14,23]. These factors underscore the importance of designing broad-spectrum biofilm dispersal agents that act on conserved regulatory or structural pathways essential for biofilm maintenance, enabling their effectiveness across a wide range of clinical isolates.

### 2.4. Evaluation of the Cytotoxicity of N-Arylimino-1,2,3-Dithiazole Derivatives ***5a***–***c*** Toward Eukaryotic Cells

**Figure 6:**
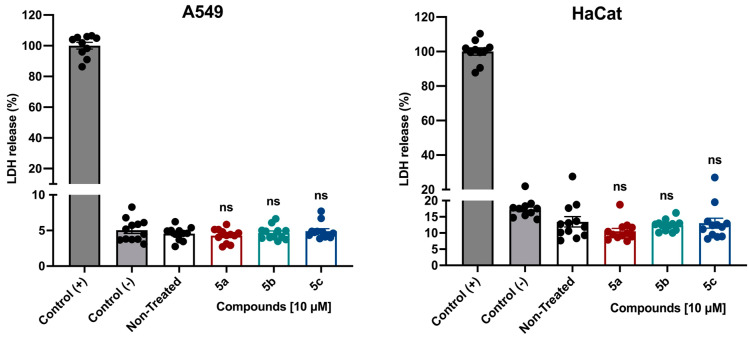
Cytotoxicity measurements on A549 and HaCat eukaryotic cells upon exposure to the *N*-arylimino-1,2,3-dithiazole-derived compounds **5a**–**c**. The cytotoxicity of the compounds **5a**, **5b**, and **5c** at 10 µM was evaluated by assessing LDH release (%) by A549 and HaCaT eukaryotic cell lines. Positive control (Triton X-100 solution was added) was considered as 100% of LDH release. Negative control was considered as the minimal LDH release in DMEM medium by A549 and HaCaT. In the non-treated condition, DMSO at 1%, used as solvent vehicle of the compounds, was added. Data are the results of the analysis of four technical replicates for each condition from at least three independent biological experiments (*n* = 3). Statistics were performed by one-way ANOVA followed by Dunnett’s multiple-comparison test. ns, not significant (*p* > 0.05).

Considering the potential use of the selected *N*-arylimino-1,2,3-dithiazole derivatives (**5a**, **5b**, and **5c**) as therapeutic adjuvants against *P. aeruginosa*-related respiratory tract and skin infections, we sought to assess their cytotoxicity towards eukaryotic cells. The A549 and HaCaT immortalized human cell lineages from alveolar basal epithelial cells and keratinocyte cells as representative models of lung and skin tissues, respectively, were used to evaluate the potential cytotoxicity at 10 µM through a Lactate Dehydrogenase (LDH) assay. LDH is an enzyme that catalyzes the reversible conversion of pyruvate to lactate in the presence of NAD+/NADH, H^+^. As an intracellular enzyme, its release into the extracellular environment is used as an indicator of cell damage. In the non-treated condition, it should be noted that LDH release by HaCat cutaneous cells (13.4%) is greater than that by A549 lung cells (4.5%) (Figure 6). The selected *N*-arylimino-1,2,3-dithiazoles possessing a quinazoline-type extended aromatic moiety did not exhibit increased cytotoxicity on the two cellular lineages (A549 and HaCat), since LDH release values were not significantly different from the non-treated conditions (Figure 6). This observation aligns with previously reported data. For instance, substituted 1,2,3-dithiazoles evaluated for their efficacy against the feline immunodeficiency virus (FIV) as a model for HIV in cells showed low cytotoxicity against feline kidney cells [43,44]. Van Horn et al. showed a limited cytotoxicity toward human cells (A549 cells) of a series of *N*^2^,*N*^4^-disubstituted quinazoline-2,4-diamines tested for their in vitro and in vivo antibacterial activity against methicillin-resistant *S. aureus* strains [45]. These compounds also showed a low cytotoxic effect on human HepG2 cells (human liver epithelial cells with hepatocellular carcinoma) [46]. In addition, it has been reported that a quinazoline derivative compound with a potent antibacterial efficacy against *P. aeruginosa* showed a low-level cytotoxicity when tested against RAW-264.7 cells (murine macrophage cells) [40].

Overall, these results demonstrate that the *N*-phenylquinazolin-4-amines **5a**, **5b**, and **5c** are not cytotoxic, at concentrations up to 10 µM, towards A549 and HaCat eukaryotic cells.

### 2.5. Effect of N-Arylimino-1,2,3-Dithiazoles ***5a***–***c*** on the Membrane Fluidity of P. aeruginosa

Bacteria use membrane fluidity to sense and respond to environmental changes. It is possible, therefore, that alterations in membrane fluidity homeostasis can trigger biofilm dispersion by modulating multiple cellular pathways. We therefore can speculate that exposure of biofilm cells to 1,2,3-dithiazole derivatives can alter membrane fluidity. These membrane perturbations may activate sensor kinases and c-di-GMP–related enzymes, leading to reduced intracellular c-di-GMP levels that promote motility and dispersal [14]. Altered fluidity may also affect transmembrane proteins involved in quorum sensing and stress responses [47,48], shifting gene expression toward dispersal and flagellar activity. Additionally, these changes may destabilize cell-matrix interactions and enhance matrix component release, facilitating cell detachment and biofilm dispersion.

**Figure 7:**
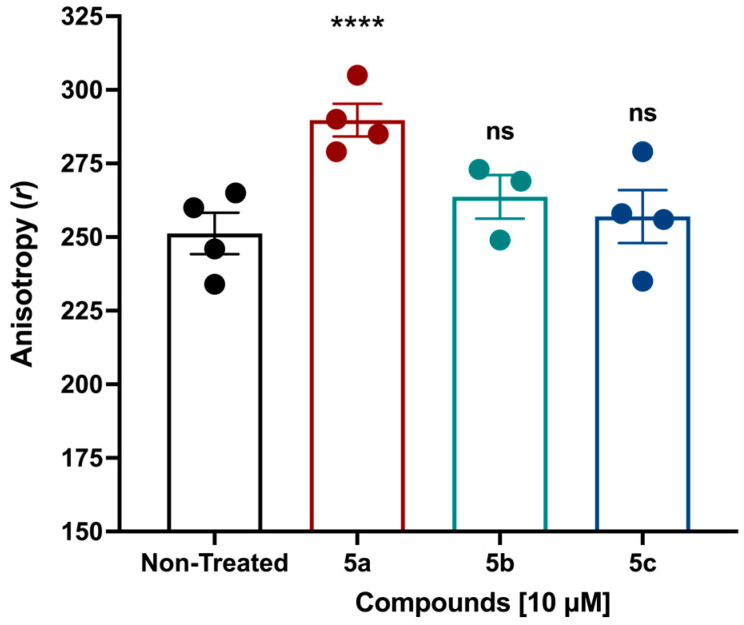
Membrane fluidity of *P. aeruginosa* exposed to imino-1,2,3-dithiazole-derived compounds. The bars represent fluorescence anisotropy values (*r*) for the PA14 strain non-treated (1% DMSO) or treated with compounds **5a**, **5b**, and **5c** for 2 h at 10 µM. DPH fluorescence was measured with an excitation wavelength of 365 nm and an emission wavelength of 425 nm. Data are the results of the analysis of means of twelve technical replicates (control condition), and four technical replicates (for each treated condition) from at least three independent biological experiments (*n* ≥ 3). Statistics were performed by one-way ANOVA followed by Dunnett’s multiple-comparison test. Values that are significantly different are indicated by asterisks as follows: ****, *p* < 0.0001; ns, not-significant (*p* > 0.05).

The dispersion of biofilms occurs following a passive or an active process [42]. Passive dispersal is related to external fluid or solid forces that physically shear the biofilm. Active dispersal refers to events triggered by the biofilm microorganisms themselves in response to environmental variations, such as nutrient starvation, low oxygen levels, or toxic by-products. In the present study, the triggering mechanism of biofilm dispersion seems to involve interactions between the compound and the bacterial membrane. The effect on membrane fluidity of the selected *N*-arylimino-1,2,3-dithiazole-derived compounds (**5a**, **5b**, and **5c**) involved in *P. aeruginosa* biofilm dispersion was assessed through fluorescence anisotropy experiments. The compound **5a** assayed at 10 µM demonstrated a significant increase of 15% in anisotropy as compared to the non-treated condition (Figure 7). Since an increased anisotropy value reflects a membrane stiffness enhancement, compound **5a** appears to decrease *P. aeruginosa* PA14 membrane fluidity. Compounds **5b** and **5c** showed a similar trend variation in anisotropy values, but not significant as compared to the control condition (non-treated PA14 strain) (Figure 7), suggesting that these two compounds slightly impact membrane fluidity. It seems that **5a** interacts with or diffuses into *P. aeruginosa* membranes more efficiently than its congeners **5b** and **5c** that trigger an alteration in membrane fluidity, which in turn may activate an envelope stress response. Further research is needed to elucidate the detailed mechanism of action of these promising dispersal agents. For instance, global multi-omics profiling approaches would be useful for identifying shifts in regulatory networks (c-di-GMP signaling), expression of matrix-degrading enzymes, metabolic reprogramming, and extracellular-matrix composition changes active at dispersal.

## 3. Materials and Methods

### 3.1. Chemistry

#### 3.1.1. General Information

All reagents were purchased from commercial suppliers and used without further purification. All reactions were monitored by thin-layer chromatography with aluminum plates (0.25 mm) precoated with silica gel 60 F254 (Merck KGaA, Darmstadt, Germany). Visualization was performed with UV light at a wavelength of 254 nm. Purifications were conducted with a flash column chromatography system (PuriFlash, Interchim, Montluçon, France) using stepwise gradients of petroleum ether (also called light petroleum) (PE) and dichloromethane (DCM) as the eluent. Melting points were measured with an SMP3 Melting Point instrument (STUART, Bibby Scientific Ltd., Roissy, France) with a precision of 1.5 °C. IR spectra were recorded with a Spectrum 100 Series FTIR spectrometer (PerkinElmer, Villebon S/Yvette, France). Liquids and solids were investigated with a single-reflection attenuated total reflectance (ATR) accessory; the absorption bands are given in cm^−1^. NMR spectra (^1^H, ^13^C, and ^19^F) were acquired at 295 K using an AVANCE 300 MHz spectrometer (Bruker, Wissembourg, France) at 300, 75, and 282 MHz. The coupling constant J was in Hz, and chemical shifts are given in ppm. Mass (ESI, EI, and field desorption (FD)) were recorded with an LCP 1er XR spectrometer (WATERS, Guyancourt, France). Mass spectrometry was performed by the Mass Spectrometry Laboratory of the University of Rouen Normandy.

#### 3.1.2. Compound Series **1a**–**h** and **5j** Were Described in Ref. [26]

Products **1i**, **1j**, and **1k** were described in refs. [49], [50], and [51], respectively. Compounds **2a**, **2b**, and **3a**–**c** have been described in ref. [52]. Compounds **2c** and **2d** were described in ref. [53] and [54], respectively. Products **4a**–**c** were described in ref. [32]. Compound **4d** was described in ref. [54], while **4e** and **4f** were described in ref. [55].

#### 3.1.3. Synthesis of 6-Aminoquinazolines (**IIIa**–**i**)

**Scheme 2:**
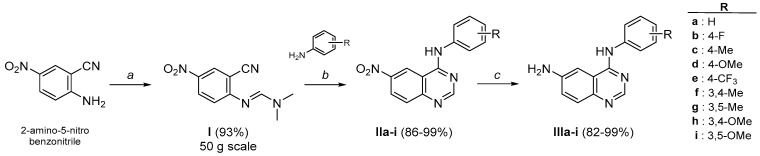
Synthesis of 6-aminoquinazolines **IIIa**–**i** (isolated yields). Conditions: (*a*) DMF-DMA (0.4 M), EtOAc (1 M), 70 °C, 30 min. (*b*) 4-substituted aniline (1.5 equiv), AcOH (1 M), 118 °C, 30 min. (*c*) Pd/C (10% *w*/*w*), HCO_2_NH_4_ (5.0 equiv), EtOH (0.2 M), reflux, 1 h.

Compounds **IIIa**–**i** were synthesized from **IIa**–**I**, obtained from (*E*)-*N*′-(2-cyano-4-nitrophenyl)-*N,N*-dimethylformimidamide **I** and 5-nitroanthranilonitrile (2-amino-5-nitrobenzonitrile), as depicted in Scheme 2 and according to previous methods [32,56,57,58,59].

Compounds in series **II** and **IIIa**–**e** and **IIIh** were previously described [50,51,52,53,54,55,56,57,58,59,60]. Their ^1^H NMR (300 MHz, CDCl_3_) spectra are in accordance with the literature data. Compounds **IIIf** and **IIIg** have CAS numbers (1481854-39-0 and 1481841-73-9, respectively) but are not described in patents or publications. Product **IIIi** has been incompletely reported in ref. [60]. These three compounds are completely characterized (see Supplementary Materials).

#### 3.1.4. Synthesis of (Z)-6-[(4-chloro-5*H*-1,2,3-dithiazol-5-ylidene)amino]-*N*-phenylquinazolin-4-amines (**5a**–**i**) (Scheme 3)

Appel Salt (1.2 eq) was added to a solution of *N*^4^-phenylquinazoline-4,6-diamine derivative (**IIIa**–**i**) 1.0 equiv), diluted in CH_2_Cl_2_ (0.3 M). The mixture was stirred at 20 °C for 1 h, then DIPEA (2.0 equiv) was added, and the resulting mixture was stirred at 20 °C for an additional hour. The resulting solution was diluted with water and extracted three times with CH_2_Cl_2_. The organic layers were washed with brine, dried over MgSO_4_, filtered, and concentrated under vacuum. The crude product was purified on silica gel by column chromatography (CH_2_Cl_2_/EtOAc 100:0 to CH_2_Cl_2_/EtOAc 0:100, *v*/*v*) to afford the desired compound.

**Scheme 3:**
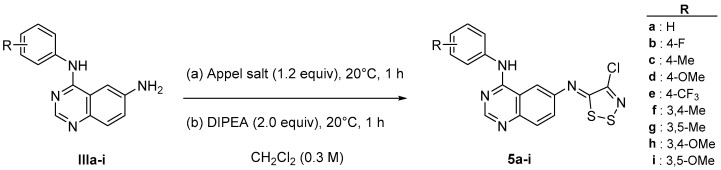
General procedure for the synthesis of compounds **5a**–**i**.

(*Z*)-6-[(4-Chloro-5*H*-1,2,3-dithiazol-5-ylidene)amino]-*N*-phenylquinazolin-4-amine (**5a**). Orange solid (0.077 g, 49%); m.p. 171–172 °C. IR (neat) *ν*_max_: 3437, 2921, 1600, 1558, 1538, 1492, 1447, 1406, 1381, 1358, 1199, 1150 cm^−1^. ^1^H NMR (400 MHz, DMSO-d_6_) *δ* 9.93 (s, 1H, N*H*), 8.62 (s, 1H, H-2), 8.41 (d, *J* = 2.2 Hz, 1H, H-5), 7.92 (d, *J* = 8.8 Hz, 3H, H-8), 7.86 (d, *J* = 7.6 Hz, 2H, H-2′, H-6′), 7.77 (dd, *J* = 8.8, 2.2 Hz, 1H, H-7), 7.40 (t, 2H, *J* = 7.6 Hz, H-3′, H-5′), 7.15 (t, *J* = 7.6 Hz, 1H, H-4′). ^13^C NMR (101 MHz, DMSO-d_6_) *δ* 161.5, 157.70, 153.9, 149.4, 146.6, 138.9, 129.48, 128.5 (2C), 125.4, 123.96, 122.4 (2C), 116.0, 112.8. HRMS (EI^+^) *m*/*z*, calcd for C_16_H_11_ClN_5_S_2_ [M+H]^+^: 372.0139, found: 372.0137.

(*Z*)-6-[(4-Chloro-5*H*-1,2,3-dithiazol-5-ylidene)amino]-*N*-(4-fluorophenyl)quinazolin-4-amine (**5b**). Yellow solid (0.241 g, 63%); m.p. 281–282 °C. IR (neat) *ν*_max_: 3443, 1610, 1575, 1540, 1507, 1423, 1225 cm^−1^. ^1^H NMR (400 MHz, DMSO-d_6_) *δ* 9.95 (s, 1H, N*H*), 8.61 (s, 1H, H-2), 8.38 (d, *J* = 2.2 Hz, 1H, H-5), 7.92–7.86 (m, 3H, H-8, H-2′, H-6′), 7.77 (dd, *J* = 8.8, 2.2 Hz, 1H, H-7), 7.29–7.20 (m, 2H, H-3′, H-5′). ^13^C NMR (101 MHz, DMSO-d_6_) *δ* 161.7, 160.4, 157.9, 157.21, 153.4, 149.7, 146.6, 134.9, 128.6, 125.8, 124.7 (d, *J* = 8.0 Hz, 2C), 115.7, 115.2 (d, *J* = 22.4 Hz, 2C), 113.0. ^19^F NMR (376 MHz, DMSO-d_6_) δ −118.37 (s, 1F). HRMS (EI^+^) *m*/*z*, calcd for C_16_H_10_ClFN_5_S_2_ [M+H]^+^: 390.0045, found: 390.0055.

(*Z*)-6-[(4-Chloro-5*H*-1,2,3-dithiazol-5-ylidene)amino]-*N*-(*p*-tolyl)quinazolin-4-amine (**5c**). Orange solid (0.164 g, 43%); m.p. 188–189 °C. IR (neat) *ν*_max_: 3442, 1565, 1537, 1513, 1419, 1401, 1382, 1358, 1199, 1125 cm^−1^. ^1^H NMR (400 MHz, DMSO-d_6_) *δ* 9.73 (s, 1H, N*H*), 8.56 (s, 1H, H-2), 8.38 (d, *J* = 2.1 Hz, 1H, H-5), 7.89 (d, *J* = 8.8 Hz, 1H, H-8), 7.73 (dd, *J* = 8.6, 3.5 Hz, 3H, H-7, H-2′, H-6′), 7.19 (d, *J* = 8.3 Hz, 2H, H-3′, H-5′), 2.30 (s, 3H, 4′-C*H*_3_). ^13^C NMR (101 MHz, DMSO-d_6_) *δ* 161.8, 158.2, 152.8, 149.9, 146.5, 135.6, 134.0, 129.0, 126.1, 123.1, 115.6, 113.2, 20.6. HRMS (EI^+^) *m*/*z*, calcd for C_17_H_13_ClN_5_S_2_ [M+H]^+^: 386.0295, found: 386.0296.

(*Z*)-6-[(4-Chloro-5*H*-1,2,3-dithiazol-5-ylidene)amino]-*N*-(4-methoxyphenyl)quinazo lin-4-amine (**5d**). Orange solid (0.182 g, 60%); m.p. 180–181 °C. IR (neat) *ν*_max_: 3342, 1570,1533, 1510, 1499, 1423, 1358, 1319, 1241, 12,081 1181, 1145, 1031 cm^−1^. ^1^H NMR (400 MHz, DMSO-d_6_) *δ* 9.73 (s, 1H, N*H*), 8.52 (s, 1H, H-2), 8.34 (d, *J* = 2.1 Hz, 1H, H-5), 7.88 (d, *J* = 8.8 Hz, 1H, H-8), 7.77–7.67 (m, 3H, H-7, H-2′, H-6′), 6.97 (d, *J* = 9.0 Hz, 2H, H-3′, H-5′), 3.77 (s, 3H, 4′-OC*H*_3_). ^13^C NMR (101 MHz, DMSO-d_6_) *δ* 162.7, 158.9, 157.5, 150.9 (d, *J* = 8.5 Hz), 146.4, 129.7 (d, *J* = 7.2 Hz), 127.6, 125.7 (d, *J* = 10.9 Hz, 2C), 123.2, 115.0, 113.9 (2C), 55.4. HRMS (EI^+^) *m*/*z*, calcd for C_17_H_13_ClN_5_OS_2_ [M+H]^+^: 402.0245, found: 402.0265.

(*Z*)-6-[(4-Chloro-5*H*-1,2,3-dithiazol-5-ylidene)amino]-*N*-(4-(trifluoromethyl)phenyl) quinazolin-4-amine (**5e**). Brown solid (0.215 g, 54%); m.p. 157–158 °C. IR (neat) *ν*_max_: 3673, 3319, 1674, 1605, 1566, 1531, 1420, 1406, 1333, 1319, 1165 cm^−1^. ^1^H NMR (400 MHz, DMSO-d_6_) *δ* 10.03 (s, 1H, N*H*), 8.69 (s, 1H, H-2), 8.42 (d, *J* = 1.9 Hz, 1H, H-5), 8.19 (d, *J* = 8.5 Hz, 2H, H-3′, H-5′), 7.96 (d, *J* = 8.8 Hz, 1H, H-8), 7.82–7.70 (m, 3H, H-7, H-2′, H-6′). ^19^F NMR (376 MHz, DMSO-d_6_) *δ* −60.23 (s, 3F). ^13^C NMR (101 MHz, DMSO-d_6_) *δ* 169.0, 161.7, 157.4, 153.7, 149.6, 147.8, 146.5, 143.0, 130.1, 126.0, 125.7, 125.5, 123.1, 121.5, 118.8, 116.1, 112.7. HRMS (EI^+^) *m*/*z*, calcd for C_17_H_10_ClF_3_N_5_S_2_ [M+H]^+^: 440.0013, found: 440.0002.

(*Z*)-6-[(4-Chloro-5*H*-1,2,3-dithiazol-5-ylidene)amino]-*N*-(3,4-dimethylphenyl) quinazolin-4-amine (**5f**). Orange solid (0.286 g, 99%); m.p. 130 °C. IR (neat) *ν*_max_: 3312, 2918, 2045, 1561, 1529, 1499, 1419, 1377, 1143, 1064, 1020 cm^−1^. ^1^H NMR (400 MHz, DMSO-d_6_) *δ* 9.68 (s, 1H, N*H*), 8.56 (s, 1H, H-2), 8.38 (d, *J* = 1.9 Hz, 1H, H-5), 7.88 (d, *J* = 8.8 Hz, 1H, H-8), 7.72 (dd, *J* = 8.8, 2.1 Hz, 1H, H-7), 7.60 (d, *J* = 5.7 Hz, 2H, H-2’, H-6’), 7.13 (d, *J* = 8.8 Hz, 1H, H-5’), 2.24 (s, 3H, C*H*_3_), 2.21 (s, 3H, C*H*_3_). ^13^C NMR (101 MHz, DMSO-d_6_) *δ* 161.9, 158.2, 152.7, 150.0, 146.5, 136.27, 135.8, 133.0, 129.5, 127.0, 126.2, 124.2, 120.6, 115.6, 113.2, 19.6, 18.9. HRMS (EI^+^) *m*/*z*, calcd for C_18_H_15_ClN_5_S_2_ [M+H]^+^: 400.0452, found: 400.0462.

(*Z*)-6-[(4-Chloro-5*H*-1,2,3-dithiazol-5-ylidene)amino]-*N*-(3,5-dimethylphenyl)quina zolin-4-amine (**5g**). Brown solid (0.166 g, 59%); m.p. 175–176 °C. IR (neat) *ν*_max_: 3359, 1618, 1575, 1538, 1498, 1425, 1400, 1383, 1355, 1317, 1189, 1148 cm^−1^. ^1^H NMR (400 MHz, DMSO-d_6_) *δ* 9.65 (s, 1H, N*H*), 8.59 (s, 1H, H-2), 8.40 (d, *J* = 1.6 Hz, 1H, H-5), 7.89 (d, *J* = 8.8 Hz, 1H, H-8), 7.73 (dd, *J* = 8.8, 1.9Hz, 1H, H-7), 7.51 (s, 2H, H-2′, H-6′), 6.77 (s, 1H, H-4′), 2.29 (s, 6H, 3′-C*H*_3_, 5′-C*H*_3_). ^13^C NMR (101 MHz, DMSO-d_6_) *δ* 161.9, 158.2, 152.7, 150.0, 146.5, 143.5, 138.0, 137.6 (s, 2C), 127.0, 126.3 (d, *J* = 8.4 Hz), 120.7 (s, 2C), 115.6, 113.1, 21.1 (s, 2C). HRMS (EI^+^) *m*/*z*, calcd for C_18_H_15_ClN_5_S_2_ [M+H]^+^: 400.0452, found: 400.0467.

(*Z*)-6-[(4-Chloro-5*H*-1,2,3-dithiazol-5-ylidene)amino]-*N*-(3,4-dimethoxyphenyl)quina zolin-4-amine (**5h**). Red solid (0.043 g, 28%); m.p. 170–171 °C. IR (neat) *ν*_max_: 3389, 1612, 1575, 1505, 1397, 1341, 1271, 1238, 1145,1026 cm^−1^. ^1^H NMR (400 MHz, DMSO-d_6_) *δ* 9.69 (s, 1H, N*H*), 8.57 (s, 1H, H-2), 8.37 (s, 1H, H-2′), 7.88 (d, *J* = 8.7 Hz, 1H, H-8), 7.79–7.66 (m, 1H, H-5), 7.49 (d, *J* = 2.2 Hz, 2H H-2′, H-6′), 7.00–6.88 (m, 1H, H-7), 3.78 (s, 6H, 3′-OC*H*_3_, 4′-OC*H*_3_). ^13^C NMR (101 MHz, DMSO-d_6_) δ 161.7, 157.9, 153.3, 149.7, 148.4 (d, *J* = 3.6 Hz, 2C), 146.5, 145.9, 131.7, 128.1, 125.7, 115.7, 115.0, 112.9, 111.7, 107.8, 55.66 (d, *J* = 19.5 Hz, 2C). HRMS (EI^+^) *m*/*z*, calcd for C_18_H_15_ClN_5_OS_2_ [M+H]^+^: 432.0350, found: 432.0343.

(Z)-6-[(4-Chloro-5*H*-1,2,3-dithiazol-5-ylidene)amino]-*N*-(3,5-dimethoxyphenyl) quinazolin-4-amine (**5i**). Orange solid (0.295 g, 70%); m.p. 128–129 °C. IR (neat) *ν*_max_: 3326, 1607, 1574, 1533, 1501, 1458, 1426, 1404, 1201, 1152, 1062 cm^−1^. ^1^H NMR (400 MHz, DMSO-d_6_) *δ* 9.66 (s, 1H, N*H*), 8.64 (s, 1H, H-2), 8.39 (d, *J* = 2.1 Hz, 1H, H-5), 7.92 (d, *J* = 8.8 Hz, 1H, H-8), 7.75 (dd, *J* = 8.8, 2.2 Hz, 1H, H-7), 7.26 (d, *J* = 2.1 Hz, 2H, H-2′, H-6′), 6.29 (t, *J* = 2.2 Hz, 1H, H-4′), 3.76 (s, 6H, 3′-OC*H*_3_, 5′-OC*H*_3_). ^13^C NMR (101 MHz, DMSO-d_6_) *δ* 161.6, 160.3 (s, 2C), 157.6, 153.6, 149.6, 146.7, 146.5, 140.6, 129.4, 125.4, 116.0, 112.6, 100.40 (s, 2C), 95.8, 55.2 (s, 2C). HRMS (EI^+^) *m*/*z*, calcd for C_18_H_15_ClN_5_OS_2_ [M+H]^+^: 432.0350, found: 432.0367.

### 3.2. Biological Work

#### 3.2.1. Bacterial Strains and Culture Conditions

The *Pseudomonas aeruginosa* PA14 strain from Harvard Medical School (Boston, MA, USA) was provided by Biomerit Research Center (Univ. Cork, Ireland). PA14 precultures were grown in 10 mL of Luria Bertani (LB) medium (BD Difco^TM^, Le Pont de Claix, France) and incubated overnight for 18 h at 37 °C in an aerobic atmosphere within a rotary shaker (Eppendorf Innova^®^ 42, Hamburg, Germany) at 180 rpm.

#### 3.2.2. Biofilm Formation and Dispersion Assays

In both the biofilm formation and dispersion assays, the cell density of an overnight culture of the PA14 strain was recorded at an OD_580 nm_ using a Thermo Spectronic spectrophotometer (Cambridge, UK), then adjusted to a value of 0.1 in LB medium containing DMSO at 1%. Next, a 96-well microtiter plate was filled with the bacterial suspension to reach a final volume of 200 µL. The conventional lids of the microtiter plates were replaced by Nunc™ Immuno TSP Lids (Thermo Fisher Scientific, Roskilde, Denmark), which allow bacterial cell adhesion and biofilm formation in the pins.

For biofilm formation assays, the tested compounds were added at the onset of the experiment at final concentrations of 10 μM, 1 μM, 0.1 μM, and 0.01 μM. Biofilm culture was carried out by incubating the microtiter plate for 24 h at 37 °C in static conditions. Then, a CV-staining assay was performed, as described below.

For biofilm dispersion assays, preformed 24 h-old biofilms grown as described above were exposed for 2 h at 37 °C by switching the lid to a challenging 96-well microtiter plate containing LB medium (DMSO 1%) supplemented with the assayed compounds at concentrations of 10 μM, 1 μM, 0.1 μM, and 0.01 μM. Subsequently, a CV-staining assay was performed, as described below.

Upon exposure to the tested compounds, the CV-staining was carried out as follows: the pins containing the residual biofilms were rinsed three times with Milli-Q water to remove the non-adhered bacterial cells. Afterwards, biofilms were stained using a solution of CV at 1% for 20 min. A further triple rinsing with Milli-Q water was performed to eliminate the excess of the CV. The biofilms adhered to pins were solubilized in a solution of acetic acid at 30% and set on a shaking plate for 5 min to detach stained cells from the pins. Finally, the residual biofilm biomass was determined by measuring the absorbance at 595 nm using the Spark 20 M multimode Microplate Reader controlled by SparkControl^TM^ software Version 2.1 (Tecan Group Ltd., Männedorf, Switzerland). At least four independent biological replicates were performed for each set of experiments.

#### 3.2.3. Monitoring of Growth Kinetics

The effect of the tested compounds on growth kinetics was monitored over the course of 24 h. In brief, planktonic PA14 cells diluted in LB medium (1% DMSO) at an OD_580 nm_ value of 0.1 containing the selected compounds at four concentrations 10 µM, 1 µM, 0.1 µM, and 0.01 µM were placed in a 96-well microtiter plate. Then, the microplate was placed in the Spark 20 M multimode Microplate Reader controlled by SparkControl^TM^ software Version 2.1 (Tecan Group Ltd., Männedorf, Switzerland), and OD was measured at 580 nm every 30 min during 24 h at 37 °C in shaking conditions. The bacterial growth curve for untreated (control condition) and treated cultures was determined by plotting the values against time.

#### 3.2.4. Cytotoxicity Assays of the Compounds

The A549 and HaCaT immortalized human cell lineages from alveolar basal epithelial and keratinocyte cells, respectively, were used to assess the cytotoxicity of the candidate compounds. The A549 and HaCat cells were cultured in a BioLite™ 250 mL flask (ThermoScientific^TM^, Roskilde, Denmark) containing Dulbecco’s Modified Eagle Medium (DMEM) supplemented with 10% of Fetal Bovine Serum (FBS) and 1% of penicillin/streptomycin antibiotics for 2–3 days, at 37 °C and 5% CO_2_. When cell density reached the state of confluence (80% of available surface), cells were rinsed by adding 2 mL of PBS 1X. After removal of PBS, cells were detached by a 2 mL trypsin treatment for 10 min, at 37 °C and 5% CO_2_. Then, 2 mL of the DMEM-supplemented medium was added to neutralize the trypsin activity, leading to a volume of 4 mL in the flask. This 4 mL volume was centrifuged, and the pellet was resuspended in 1 mL of DMEM-supplemented medium. The cells were diluted in 39 mL of DMEM supplemented medium. One mL of eukaryotic cell suspension was placed in each well of a 24-well microplate and incubated for 2–3 days at 37 °C and 5% CO_2_. When the confluence stage had been reached, the medium was removed and replaced by 500 µL of DMEM medium containing each compound at 10 µM. The A549 and HaCat cells were then exposed for 2 h at 37 °C and 5% CO_2_. For the negative control condition, 500 µL of DMEM medium was added to the cells. For the positive control condition, 500 µL of Triton X100 was added to the cells. Subsequently, a Lactate Dehydrogenase (LDH) assay (CyQUANT^TM^ LDH Cytotoxicity Assay, Invitrogen^TM^, Thermo Fischer Scientific, Willow Creek Road, Eugene, OR, USA) was carried out following the manufacturer’s instructions. Briefly, 50 µL of supernatant was collected from each well and transferred to a 96-well microtiter plate. Next, 50 µL of Start Solution was added to each well of the microtiter plate and incubated in the dark for 30 min. The Start Solution contains yellow tetrazolium salt, which is converted by NADH into a red formazan-class dye. The amount of red formazan is directly proportional to the amount of LDH in the culture, which is also directly proportional to the number of damaged or dead cells in the culture. To stop the reaction, 50 µL of Stop Solution was added to each well. Finally, the absorbance was measured at 490 nm and 680 nm (background noise) wavelengths using the Spark 20 M multimode Microplate Reader controlled by SparkControl^TM^ software Version 2.1 (Tecan Group Ltd., Männedorf, Switzerland).

#### 3.2.5. Pyocyanin Quantification Assays

To evaluate pyocyanin production, a cell suspension of the PA14 strain prepared in LB medium containing 1% DMSO and adjusted to a final OD_580 nm_ value of 0.1 was placed in a 96-well microtiter plate. The tested compounds were added at a final concentration of 10 µM and the microtiter plate was incubated for 24 h at 37 °C under shaking conditions (180 rpm). Then, both the treated and non-treated cultures were harvested and centrifuged for 5 min at 7500× *g*, at room temperature. To extract the pyocyanin pigment, the supernatant was collected and mixed with one volume of chloroform. The mixture was vortexed for ten seconds and centrifuged for 5 min at 7500× *g* to separate the aqueous and the organic phases. The aqueous phase was discarded. Then, a half volume of HCl 0.5 N was added to the organic phase (blue layer). The mixture was vortexed for ten seconds and centrifuged for 5 min at 7500× *g*. The upper phase (pink layer) was collected and transferred to a microtiter plate. Finally, the absorbance was measured at 520 nm using the Spark 20 M multimode Microplate Reader controlled by SparkControl^TM^ software Version 2.1 (Tecan Group Ltd., Männedorf, Switzerland), and the data were normalized for bacterial cell density (OD_580 nm_).

#### 3.2.6. Fluorescence Anisotropy Assays

Cells of the PA14 strain resuspended in LB medium (DMSO at 1%) containing 10 µM of the tested compounds were placed in a 96-well microtiter plate at a final OD_580 nm_ value of 0.1. The microtiter plate was incubated for 24 h at 37 °C in a rotary shaker (180 rpm). Then, non-treated (control condition) and treated bacterial cultures were harvested and centrifuged for 5 min at 7500× *g* (room temperature). The supernatant was discarded, and the pellet was washed twice using a solution of MgSO_4_^2−^ at 10 mM. Bacterial suspensions were adjusted to a final OD_580 nm_ value of 0.1 for fluorescence anisotropy measurements. Next, 1 µL of 1,6-diphenyl-1,3,5-hexatriene (DPH) solution was added to non-treated and treated samples at a final concentration of 4 µM and incubated at 37 °C for 1 h in the dark. Finally, fluorescence anisotropy was determined at an excitation wavelength of 365 nm and an emission wavelength of 425 nm using the Spark 20 M multimode Microplate Reader controlled by SparkControl^TM^ software Version 2.1 (Tecan Group Ltd., Männedorf, Switzerland).

#### 3.2.7. Statistical Analyses

Statistical significance was evaluated using GraphPad Prism software version 10.2.3. The data were statistically analyzed using ordinary one-way analysis of variance (ANOVA) followed by Dunnett’s multiple comparison test, to calculate *p*-values. Experiments were performed with at least three biological independent replicates, and results were displayed as means ± SEMs (standard error of the means).

## 4. Conclusions

The current study allowed the identification of *N*-arylimino-1,2,3-dithiazole derivatives that induce a significant dispersion activity on preformed *P. aeruginosa* biofilms. It may be important to test these compounds on clinical isolates to validate their broad-spectrum dispersal effects. These derivatives do not appear to be bactericidal towards planktonic cells and are not cytotoxic towards two eukaryotic human cell lines (A549 and HaCat). Moreover, these compounds do not increase the virulence of *P. aeruginosa* through the production of pyocyanin. The quinazoline derivative **5a** exhibited reduced membrane fluidity, suggesting a preliminary insight into the potential mode of action associated with its dispersal effect. Further investigations are now required to comprehensively elucidate the mode of action of these promising compounds. Altogether, these findings highlight the potential of quinazoline derivatives as safe and effective agents for in vitro biofilm control. However, experimental models that accurately replicate the physiologically relevant conditions of clinical biofilms, including ex vivo and in vivo systems, would enable a more precise investigation of the biofilm dispersal effects of quinazoline-derivatives.

## Figures and Tables

**Table 1:** *P. aeruginosa* biofilm dispersal upon 2 h exposure to *N*-arylimino-1,2,3-dithiazole derivatives (series **1a**–**l**, **2a**–**e**, and **3a**–**c**).

	Residual Biofilm (%) ^$^			
Compound	10 μM	1 μM	0.1 μM	0.01 μM
**1a**	95.2	102.2	96.6	103.6
**1b**	113.8	99.5	96.3	87.6
**1c**	94.5	95.8	91.0	88.4
**1d**	94.8	101.6	103.1	110.1
**1e**	84.1	91.1	77.7	85.8
**1f**	91.9	98.0	92.2	98.0
**1g**	103.7	89.9	111.1	78.8
**1h**	76.3	77.7	89.6	89.1
**1i**	81.1	87.1	89.9	90.7
**1j**	65.3	79.7	90.5	80.3
**1k**	87.3	96.1	89.2	92.3
**1l**	73.1	73.8	82.8	93.4
**2a**	71.6	71.5	100.6	96.1
**2b**	83.6	82.4	89.7	95.7
**2c**	103.5	113.5	108.5	97.5
**2d**	94.4	96.3	91.8	95.2
**2e**	77.9	106.6	89.5	97.1
**3a**	69.1	55.1	91.4	90.2
**3b**	71.1	86.0	101.4	100.3
**3c**	121.3	108.9	98.0	102.5

^$^ Data are the results of the analysis of means of twelve technical replicates (control condition), and two technical replicates (for each treated condition) from at least four independent biological experiments (*n* = 4).

**Table 2:** *P. aeruginosa* biofilm dispersal upon 2 h exposure to quinazolinones (**4a**–**f**) and quinazolines (**5a**–**j**).

	Residual Biofilm (%) ^$^			
Compound	10 μM	1 μM	0.1 μM	0.01 μM
**4a**	78.3	97.8	94.9	94.0
**4b**	85.0	106.6	96.5	109.7
**4c**	85.4	93.9	88.9	88.4
**4d**	90.4	90.7	93.2	102.5
**4e**	87.2	97.5	86.4	95.1
**4f**	86.1	91.2	104.5	104.4
**5a**	43.5	78.0	68.5	81.6
**5b**	56.5	107.3	102.5	107.3
**5c**	45.0	113.0	110.2	111.3
**5d**	80.1	83.7	91.9	89.3
**5e**	89.1	95.6	90.9	92.5
**5f**	87.5	83.3	84.4	90.6
**5g**	119.2	108.0	103.3	101.8
**5h**	81.3	91.2	88.3	89.3
**5i**	106.1	117.9	114.4	112.5
**5j**	74.4	128.6	93.4	85.4

^$^ Data are the results of the analysis of means of twelve technical replicates (control condition), and two technical replicates (for each treated condition) from at least four independent biological experiments (*n* = 4).

## Data Availability

Data are contained within the article and Supplementary Materials.

## Supplementary Materials

The following supporting information can be downloaded at https://www.mdpi.com/article/10.3390/ph18111733/s1. Scheme S1. Synthesis of 6-aminoquinazolines IIIa-i (isolated yields). Table S1. Individual and overall yields of the multistep synthesis of amines IIIa-i, and compounds 5a-i.
