# Supplementary material for: Quinazoline-Derivatives of Imino-1,2,3-Dithiazoles Promote Biofilm Dispersion of Pseudomonas aeruginosa
Source: Pharmaceuticals (Basel). 2025 Nov 14;18(11):1733. doi: 10.3390/ph18111733 (PMC12655343; doi:10.3390/ph18111733)

# Supplementary Materials

## Quinazoline-derivatives of imino-1,2,3-dithiazoles promote biofilm dispersion of *Pseudomonas aeruginosa*

**Mathieu Gonzalez [1,2], Anne-Sophie Tareau [1,2], Daphnée de Crozals [3], Corentin Layec [3], Nathan Broudic [3], Magalie Barreau [1,2], Adrien Forge [1,2], Olivier Lesouhaitier [1,2], Corinne Fruit [3], Sylvie Chevalier [1,2], Thierry Besson [3,*] and Ali Tahrioui [1,2,*]**

[1] Univ Rouen Normandie, Université Caen Normandie, Normandie Univ, CBSA UR 4312, F-76000 Rouen, France; mathieu.gonzalez@univ-rouen.fr (M.G.); anne-sophie.tareau@univ-rouen.fr (A.-S.T.); magalie.barreau@univ-rouen.fr (M.B.); adrien.forge@univ-rouen.fr (A.F.); olivier.lesouhaitier@univ-rouen.fr (O.L.); sylvie.chevalier@univ-rouen.fr (S.C.)

[2] Plateforme de Sécurité Sanitaire Ébroïcienne (PS$^2$E), Univ Rouen Normandie, Normandie Univ, CBSA UR 4312, F-76000 Rouen, France

[3] Univ Rouen Normandie, INSA Rouen Normandie, Univ Caen Normandie, ENSICAEN, CNRS, Institut CARMeN UMR 6064, F-76000 Rouen, France; daphnee.de-crozals@univ-rouen.fr (D.d.C.); corentin.layec@universite-paris-saclay.fr (C.L.); nathan.broudic@u-paris.fr (N.B.); corinne.fruit@univ-rouen.fr (C.F.)

* Correspondence: thierry.besson@univ-rouen.fr (T.B.); ali.tahrioui@univ-rouen.fr (A.T.)

### Table of contents

| | Pages |
|---|---|
| **General information** | **S2** |
| **Synthesis of 6-aminoquinazolines (IIIa-i) and their 6-nitroquinazoline precursors (IIa-i)** | **S3-S7** |
| **1H and 13C of new compounds** | **S8-S23** |

## General Information

All reagents were purchased from commercial suppliers and used without further purification. All reactions were monitored by thin-layer chromatography with aluminum plates (0.25 mm) precoated with silica gel 60 F254 (Merck KGaA, Darmstadt, Germany). Visualization was performed with UV light at a wavelength of 254 nm.

Purifications were conducted with a flash column chromatography system (PuriFlash, Interchim, Montluçon, France) using stepwise gradients of petroleum ether (also called light petroleum) (PE) and dichloromethane (DCM) as the eluent.

Melting points were measured with an SMP3 Melting Point instrument (STUART, Bibby Scientific Ltd., Roissy, France) with a precision of 1.5 °C.

IR spectra were recorded with a Spectrum 100 Series FTIR spectrometer (PerkinElmer, Villebon S/Yvette, France). Liquids and solids were investigated with a single-reflection attenuated total reflectance (ATR) accessory; the absorption bands are given in $cm^{-1}$.

NMR spectra ($^{1}H$, $^{13}C$ and $^{19}F$) were acquired at 295 K using an AVANCE 300 MHz spectrometer (Bruker, Wissembourg, France) at 300, 75 and 282 MHz. Coupling constant J was in Hz and chemical shifts are given in ppm. Mass (ESI, EI and field desorption (FD)) were recorded with an LCP 1er XR spectrometer (WATERS, Guyancourt, France).

Mass spectrometry was performed by the Mass Spectrometry Laboratory of the University of Rouen.

Most of the compounds in series **II** and **III** were previously described in academic works; their physicochemical characterization was limited to $^{1}H$ NMR (300 MHz, $CDCl_3$) and comparison with literature data [39-42]. Newly synthesized compounds (**II**g, **IIIf**, **IIIg** and **IIIi**) are fully characterized below).

**Synthesis of 6-aminoquinazolines (IIIa-i) and their 6-nitroquinazoline precursors (IIa-i)**

Compounds **IIIa-i** were synthesized from **IIa-i,** themselves obtained from (*E*)-*N*'-(2-cyano-4-nitrophenyl)-*N,N*-dimethylformimidamide **I** and 5-nitroanthranilonitrile (2-amino-5-nitrobenzonitrile) as depicted in Scheme 1 and according previous methods [32, 56-59] in the main text

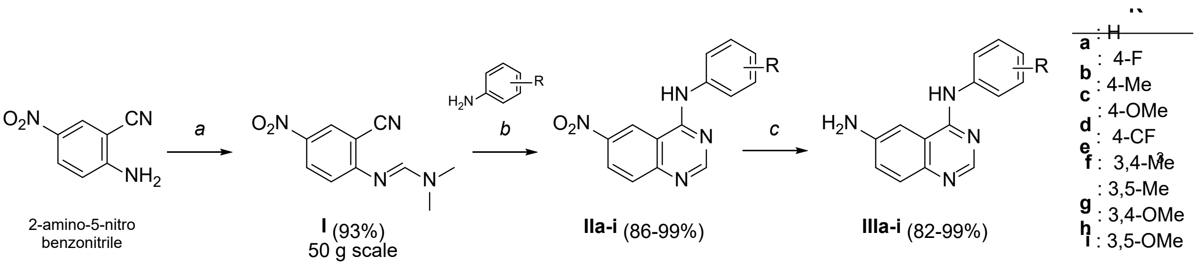


**Scheme S1.** Synthesis of 6-aminoquinazolines **IIIa-i** (isolated yields). Conditions: (a) DMFDMA (0.4 M), EtOAc (1 M), 70 °C, 30 min. (b) 4-substituted aniline (1.5 equiv), AcOH (1 M), 118 °C, 30 min. (c) Pd/C (10% *w*/*w*), $HCO_2NH_4$ (5.0 equiv), EtOH (0.2 M), reflux, 1 h. Details are given below.

*Step a.* A suspension of 2-amino-5-nitrobenzonitrile (50.0 g, 0.31 mol) in dimethylformamide dimethyl acetal (102 mL, 0.76 mol) was heated for 30 min at reflux. The resulting mixture was cooled to room temperature and refrigerated overnight. The orange precipitate formed was filtered, washed with ethyl ether, and dried to give the desired product.

*N'-(2-Cyano-4-nitrophenyl)-N,N-dimethylformimidamide (**I**)*

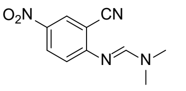

Orange solid (62 g, 93%). $^1$H NMR (400 MHz, DMSO-*d6*) $\delta$ 8.42 (s, 1H), 8.24 (m, 2H), 7.34 (d, J = 9.0 Hz, 1H), 3.16 (s, 3H), 3.07 (s, 3H).

*Step b, Synthesis of **IIa-I**, general procedure.* To a stirred solution of *N'*-(2-Cyano-4-nitrophenyl)-*N,N*-dimethylformamidine **I** (1.0 eq) in acetic acid (1 M), the appropriate amine (1.5 eq) was added. The resulting mixture was heated for 30 min at reflux. After completion of the reaction, the reaction mixture was cooled to 0°C and distilled water (50 mL) was added and shaken until the precipitates appeared. The precipitate was filtered and washed with distilled water. The resulting yellow solid was dissolved in ethanol and evaporated multiple times to remove any remaining water. For derivatives that did not precipitate upon aqueous quenching, the reaction mixture was diluted in $CH_2Cl_2$ and neutralized with a saturated solution of $NaHCO_3$. The organic layer was separated, washed twice with brine, dried over magnesium sulfate ($MgSO_4$), and concentrated under reduced pressure to afford the desired product **II a-i**.

*6-Nitro-N-phenylquinazolin-4-amine **IIa***

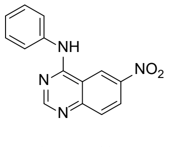


Light yellow solid (1.07 g, 94%). $^1$H NMR (400 MHz, DMSO-*d6*) $\delta$ 10.42 (s, 1H), 9.68 (d, *J* = 2.4 Hz, 1H), 8.71 (s, 1H), 8.56 (dd, *J* = 9.2, 2.4 Hz, 1H), 7.94 (d, *J* = 9.2 Hz, 1H), 7.86 – 7.81 (m, 2H), 7.44 (dd, *J* = 10.7, 5.2 Hz, 2H), 7.20 (t, *J* = 7.4 Hz, 1H).

*N-(4-Fluorophenyl)-6-nitroquinazolin-4-amine **IIb***

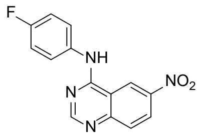


Yellow solid (1.55 g, quantitative yield). $^1$H NMR (400 MHz, DMSO-*d6*) $\delta$ 10.51 (s, 1H), 9.64 (d, *J* = 2.4 Hz, 1H), 8.69 (s, 1H), 8.56 (dd, *J* = 9.2, 2.4 Hz, 1H), 7.93 (d, *J* = 9.2 Hz, 1H), 7.88 – 7.77 (m, 2H), 7.34 – 7.21 (m, 2H).

*6-Nitro-N-(p-tolyl)quinazolin-4-amine **IIc***

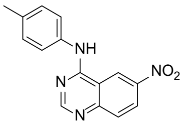


Orange solid (1.28 g, quantitative yield). $^1$H NMR (400 MHz, DMSO-*d6*) $\delta$ 10.41 (s, 1H), 9.66 (d, *J* = 2.4 Hz, 1H), 8.67 (s, 1H), 8.55 (dd, *J* = 9.2, 2.4 Hz, 1H), 7.92 (d, J = 9.2 Hz, 1H), 7.70 (d, *J* = 8.4 Hz, 2H), 7.24 (d, *J* = 8.2 Hz, 2H), 2.33 (s, 3H).

*N-(4-Methoxyphenyl)-6-nitroquinazolin-4-amine **IId***

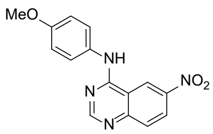


Light brown solid (1.34 g, 99%). $^1$H NMR (400 MHz, DMSO-*d6*) $\delta$ 10.39 (s, 1H), 9.62 (d, *J* = 2.4 Hz, 1H), 8.63 (s, 1H), 8.53 (dd, *J* = 9.2, 2.4 Hz, 1H), 7.90 (d, *J* = 9.2 Hz, 1H), 7.75 – 7.61 (m, 2H), 7.04 – 6.94 (m, 2H), 3.78 (s, 3H).

*6-Nitro-N-[4-(trifluoromethyl)phenyl]quinazolin-4-amine **IIe***

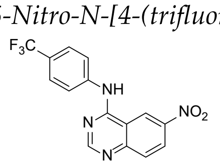


Yellow solid (1.53 g, quantitative yield). $^1$H NMR (400 MHz, DMSO-*d6*) $\delta$ 10.29 (s, 1H), 9.71 (m, 1H), 8.82 (s, 1H), 8.60 (dd, *J* = 9.2, 2.4 Hz, 1H), 8.15 (d, *J* = 8.2 Hz, 2H), 7.99 (d, *J* = 9.2 Hz, 1H), 7.79 (t, *J* = 8.2 Hz, 2H).

*N-(3,4-Dimethylphenyl)-6-nitroquinazolin-4-amine* ***IIf***

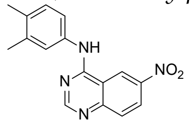


Yellow solid (779 mg, 89%). $^{1}$H NMR (400 MHz, DMSO-*d6*) $\delta$ 10.36 (s, 1H), 9.66 (d, *J* = 2.3 Hz, 1H), 8.67 (s, 1H), 8.54 (dd, *J* = 9.2, 2.4 Hz, 1H), 7.91 (d, *J* = 9.2 Hz, 1H), 7.55 (s, 2H), 7.22 – 7.15 (m, 1H), 2.25 (d, 6H).

*N-(3,5-Dimethylphenyl)-6-nitroquinazolin-4-amine* ***IIg***

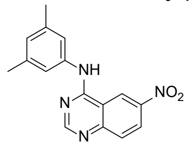


Yellow solid (600 mg, 95%); m.p. 199-200 °C. IR (neat) $\nu_{max}$: 3406.66, 1616.52, 1569.39, 1537.76, 1484.97, 1404.46, 1357.41, 1323.99, 1219.81, 1112.59, 892.13, 839.50, 809.68, 746.22, 686.28, 587.34, 537.80, 474.09 cm$^{-1}$. $^{1}$H NMR (400 MHz, DMSO-d6) δ 10.32 (s, 1H), 9.67 (d, J = 2.4 Hz, 1H), 8.71 (s, 1H), 8.55 (dd, J = 9.2, 2.4 Hz, 1H), 7.92 (d, J = 9.2 Hz, 1H), 7.47 (s, 2H), 6.84 (s, 1H), 2.31 (s, 6H). $^{13}$C NMR (101 MHz, DMSO-d6) δ 157.8, 153.1, 144.5, 138.7, 138.2, 137.5 (s, 2C), 129.5, 126.6, 126.1, 120.9, 120.6 (s, 2C), 120.2, 21.1 (s, 2C). HRMS (EI+) m/z, calcd for $C_{16}H_{15}N_4O_2$ [M+H]$^+$: 295.1195, found: 295.1193.

*N-(3,4-Dimethoxyphenyl)-6-nitroquinazolin-4-amine* ***IIh***

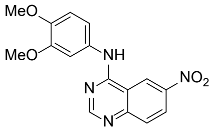


Orange solid (1.45 g, 97%). $^{1}$H NMR (400 MHz, DMSO-*d6*) $\delta$ 10.35 (s, 1H), 9.64 (d, *J* = 2.4 Hz, 1H), 8.68 (s, 1H), 8.55 (dd, *J* = 9.2, 2.4 Hz, 1H), 7.92 (d, *J* = 9.2 Hz, 1H), 7.50 – 7.33 (m, 2H), 7.01 (d, *J* = 8.6 Hz, 1H), 3.79 (s, 3H), 3.78 (s, 3H).

*N-(3,5-Dimethoxyphenyl)-6-nitroquinazolin-4-amine* ***IIi***

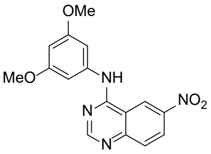


Yellow solid (1.29 g, 86%). $^{1}$H NMR (400 MHz, DMSO-*d6*) $\delta$ 10.31 (s, 1H), 9.67 (d, *J* = 1.9 Hz, 1H), 8.75 (s, 1H), 8.56 (dd, *J* = 9.2, 2.4 Hz, 1H), 7.94 (d, *J* = 9.2 Hz), 7.18 (s, 2H), 6.35 (t, 1H), 3.78 (s, 6H).

*Step c, synthesis of **IIIa-i**.* A solution of nitro compound **II** (1.0 equiv), ammonium formate (5.0 equiv), and palladium over charcoal (10% wt/wt) in EtOH (0.2 M) was heated at reflux for 30 min. The mixture was then filtered over Celite, and the solvent was evaporated. The resulting solution was diluted with EtOAc and neutralised with a solution of $NaHCO_3$ sat. The aqueous phase was extracted 3 times with EtOAc. The organic phases were washed with brine, dried over $MgSO_4$, filtered, and concentrated under reduced pressure to obtain the desired product. Some derivatives required additional purification by flash chromatography on silica gel ($CH_2Cl_2$/MeOH, 9:1, v/v).

*$N^4$-Phenylquinazoline-4,6-diamine* ***IIIa***

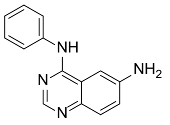


Yellow solid (222 mg, 99%). $^1$H NMR (400 MHz, DMSO-*d6*) *δ* 9.32 (s, 1H), 8.31 (s, 1H), 7.85 (dd, *J* = 8.6, 1.0 Hz, 2H), 7.52 (d, *J* = 8.8 Hz, 1H), 7.35 (dd, *J* = 9.3, 6.6 Hz, 3H), 7.23 (dd, *J* = 8.9, 2.4 Hz, 1H), 7.06 (t, *J* = 7.4 Hz, 1H), 5.56 (s, 2H).

*$N^4$-(4-Fluorophenyl)quinazoline-4,6-diamine* ***IIIb***

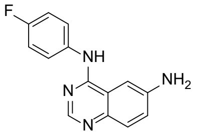


Yellow solid (251 mg, quantitative yield). $^1$H NMR (400 MHz, DMSO-*d6*) *δ* 9.37 (s, 1H), 8.29 (s, 1H), 7.90 – 7.78 (m, 2H), 7.52 (d, *J* = 8.8 Hz, 1H), 7.33 (d, *J* = 2.3 Hz, 1H), 7.26 – 7.15 (m, 3H,), 5.57 (s, 2H).

*$N^4$-(p-Tolyl)quinazoline-4,6-diamine* ***IIIc***

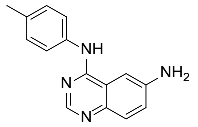


Yellow solid (249 mg, quantitative yield). $^1$H NMR (400 MHz, DMSO-*d6*) *δ* 9.24 (s, 1H), 8.28 (s, 1H), 7.71 (d, *J* = 8.4 Hz, 2H), 7.50 (d, *J* = 8.8 Hz, 1H), 7.34 (d, *J* = 2.3 Hz, 1H), 7.22 (dd, *J* = 8.9, 2.4 Hz, 1H), 7.15 (d, *J* = 8.2 Hz, 2H), 5.53 (s, 2H), 2.29 (s, 3H).

*$N^4$-(4-Methoxyphenyl)quinazoline-4,6-diamine* ***IIId***

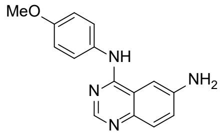


Orange solid (449 mg, quantitative yield). $^1$H NMR (400 MHz, DMSO-*d6*) *δ* 9.22 (s, 1H), 8.24 (s, 1H), 7.69 (d, *J* = 9.0 Hz, 2H), 7.49 (d, *J* = 8.8 Hz, 1H), 7.32 (d, *J* = 2.3 Hz, 1H), 7.21 (dd, *J* = 8.8, 2.3 Hz, 1H), 6.93 (d, *J* = 9.0 Hz, 2H), 5.51 (s, 2H), 3.76 (s, 3H).

*$N^4$-[4-(Trifluoromethyl)phenyl]quinazoline-4,6-diamine* ***IIIe***

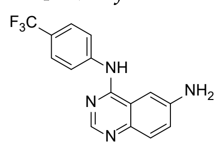


Yellow solid (273 mg, quantitative yield). $^1$H NMR (400 MHz, DMSO-*d6*) *δ* 9.67 (s, 1H), 8.40 (s, 1H), 8.15 (m, 2H), 7.83 – 7.61 (m, 2H), 7.57 (d, *J* = 8.8 Hz, 1H), 7.39 (d, *J* = 1.8 Hz, 1H), 7.28 (dd, *J* = 8.9, 1.9 Hz, 1H), 5.67 (s, 2H).

*$N^4$-(3,4-Dimethylphenyl)quinazoline-4,6-diamine* ***IIIf***

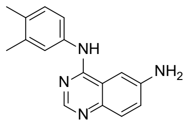


Brown solid (359 mg, 99%); m.p. 217-218 °C. IR (neat) $\nu_{max}$: 3156.54, 1596.78, 1567.08, 1530.74, 1500.60, 1422.43, 1305.63, 1215.37, 1129.99, 890.30, 852.70, 818.76, 772.05, 580.86, 533.58, 476.01 cm$^{-1}$. $^1$H NMR (400

MHz, DMSO-d6) δ 9.18 (s, 1H), 8.29 (s, 1H), 7.58 (s, 2H), 7.51 (d, J = 8.8 Hz, 1H), 7.36 (d, J = 2.2 Hz, 1H), 7.22 (dd, J = 8.9, 2.3 Hz, 1H), 7.10 (d, J = 7.9 Hz, 1H), 5.52 (s, 2H), 2.23 (s, 3H), 2.20 (s, 3H). $^{13}$C NMR (101 MHz, DMSO-d6) δ 156.2, 150.1, 147.1, 142.5, 137.6, 135.8, 130.7, 129.6, 128.6, 123.4, 123.2, 119.5, 116.6, 101.2, 19.7, 18.8. HRMS (EI+) m/z, calcd for $C_{16}H_{17}N_4$ [M+H]$^+$: 265.1453, found: 265.1459.

*N$^4$-(3,5-Dimethylphenyl)quinazoline-4,6-diamine* ***IIIg***

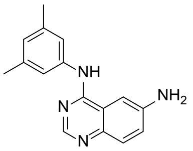


Yellow product (427 mg, quantitative yield), m.p. 148-149 °C. IR (neat) $\nu_{max}$: 3295.42, 1567.84, 1533.39, 1508.79, 1407.68, 1306.82, 1268.30, 1170.01, 1076.05, 896.93, 829.49, 693.15, 620.91, 530.77, 437.88 cm$^{-1}$. $^1$H NMR (400 MHz, DMSO-d6) δ 9.16 (s, 1H), 8.29 (s, 1H), 7.56 (d, J = 8.8 Hz, 1H), 7.50 (s, 2H), 7.36 (d, J = 2.3 Hz, 1H), 7.23 (dd, J = 8.8, 2.3 Hz, 1H), 7.09 (s, 1H), 5.52 (s, 2H), 2.23 (s, 6H). $^{13}$C NMR (101 MHz, DMSO-d6) δ 156.1, 150.0, 147.2, 142.6, 139.7, 137.2 (s, 2C), 128.6, 124.5, 123.5, 119.55 (s, 2C), 116.7, 101.2, 21.2 (s, 2C). HRMS (EI+) m/z, calcd for $C_{16}H_{1}7N_4$ [M+H]$^+$: 265.1453, found: 265.1455.

*N$^4$-(3,4-Dimethoxyphenyl)quinazoline-4,6-diamine* ***IIIh***

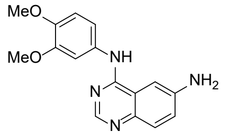


Yellow solid (219 mg, 82%). $^1$H NMR (400 MHz, DMSO-*d6*) *δ* 9.18 (s, 1H), 8.27 (s, 1H), 7.49 (dd, *J* = 8.3, 5.6 Hz, 2H), 7.42 (dd, *J* = 8.7, 2.4 Hz, 1H), 7.33 (d, *J* = 2.3 Hz, 1H), 7.21 (dd, *J* = 8.9, 2.3 Hz, 1H), 6.94 (d, *J* = 8.7 Hz, 1H), 5.51 (s, 2H), 3.77 (s, 3H), 3.75 (s, 3H).

*N$^4$-(3,5-Dimethoxyphenyl)quinazoline-4,6-diamine* ***III*i**

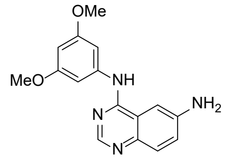


Light yellow solid (445 mg, 98%); m.p. 109-110 °C. IR (neat) $\nu_{max}$: 3329.48, 1609.85, 1569.25, 1535.68, 1509.74, 1478.51, 1413.89, 1343.94, 1269.28, 1201.06, 1152.04, 1054.35, 947.20, 902.28, 831.84, 786.01, 675.92, 631.42, 529.07 cm$^{-1}$. $^1$H NMR (400 MHz, DMSO-*d6*) *δ* 9.23 (s, 1H), 8.38 (s, 1H), 7.54 (d, *J* = 8.8 Hz, 1H), 7.37 (d, *J* = 2.3 Hz, 1H), 7.25 (dd, *J* = 9.2, 2.3 Hz, 3H), 6.24 (t, *J* = 2.2 Hz, 1H), 5.58 (s, 2H), 3.76 (s, 6H). $^{13}$C NMR (101 MHz, DMSO-*d6*) *δ* 160.3 (s, 2C), 155.9, 149.8, 147.3, 142.6, 141.7, 128.7, 123.7, 116.8, 101.0 (s, 2C), 99.7, 94.7, 55.1 (s, 2C). HRMS (EI+) *m/z*, calc for $C_{16}H_{17}N_4O_2$ [M+H]$^+$: 297.1352, found: 297.1353.

*Method (d)* was performed as described in the main text.

**Table S1.** Individual and overall yields of the multistep synthesis of amines **IIIa-i,** and compounds **5a-i**

| R | yield in **II** (%) | yield in **III** (%) | Yield in **5** (%) | Overall yield (%) | Product Series |
|---|---|---|---|---|---|
| H | 94 | 99 | 49 | 84 | **a** |
| 4-F | Quant. | Quant. | 63 | 89 | **b** |
| 4-$CH_3$ | Quant. | Quant. | 43 | 84 | **c** |
| 4-$OCH_3$ | 99 | Quant. | 60 | 88 | **d** |
| 4-$CF_3$ | Quant. | Quant. | 54 | 87 | **e** |
| 3,4-$CH_3$ | 89 | 99 | 99 | 95 | **f** |
| 3,5-$CH_3$ | 95 | Quant. | 59 | 87 | **g** |
| 3,4-$OCH_3$ | 97 | 82 | 28 | 75 | **h** |
| 3,5-$OCH_3$ | 86 | 98 | 70 | 87 | **i** |

## 1. $^{1}H$, $^{13}C$, and $^{19}F$ NMR spectra of new compounds

*N-(3,5-Dimethylphenyl)-6-nitroquinazolin-4-amine* **IIg**

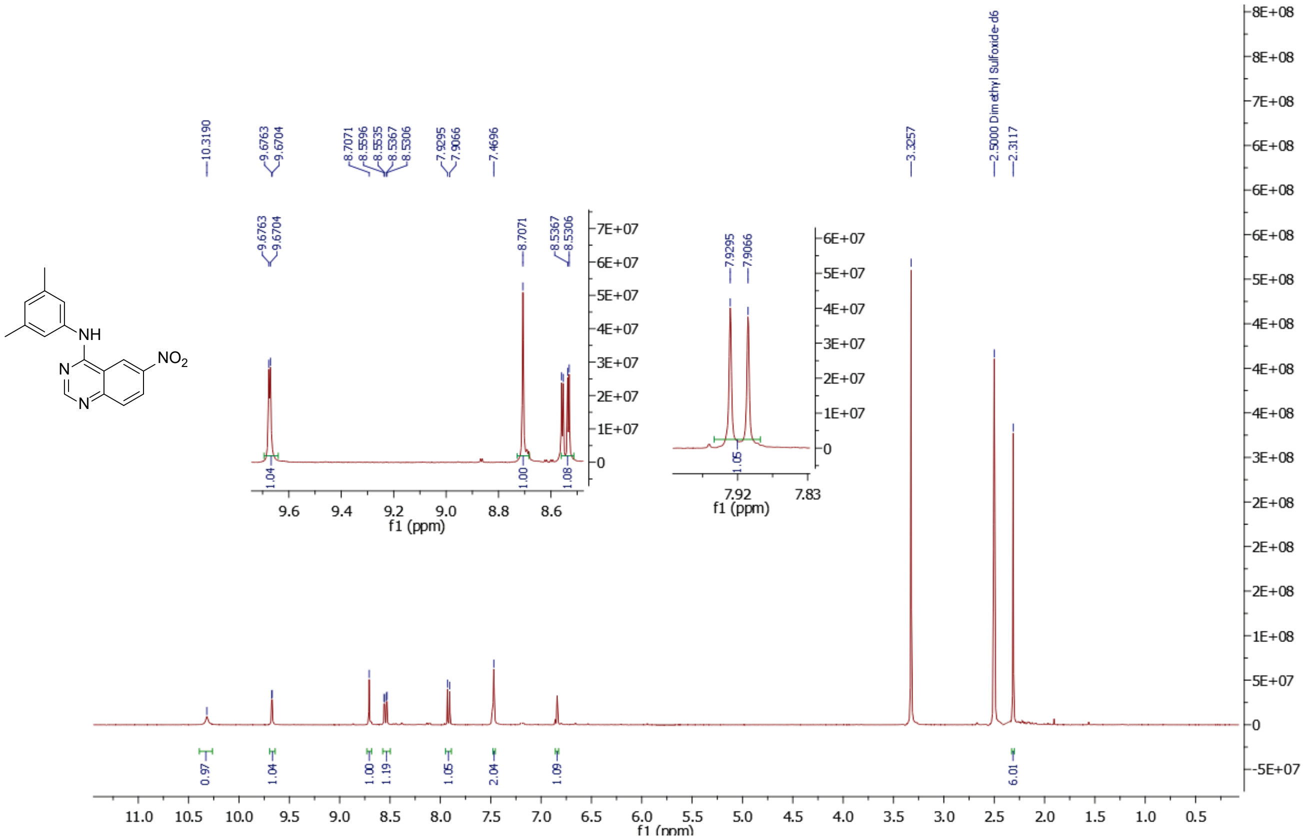


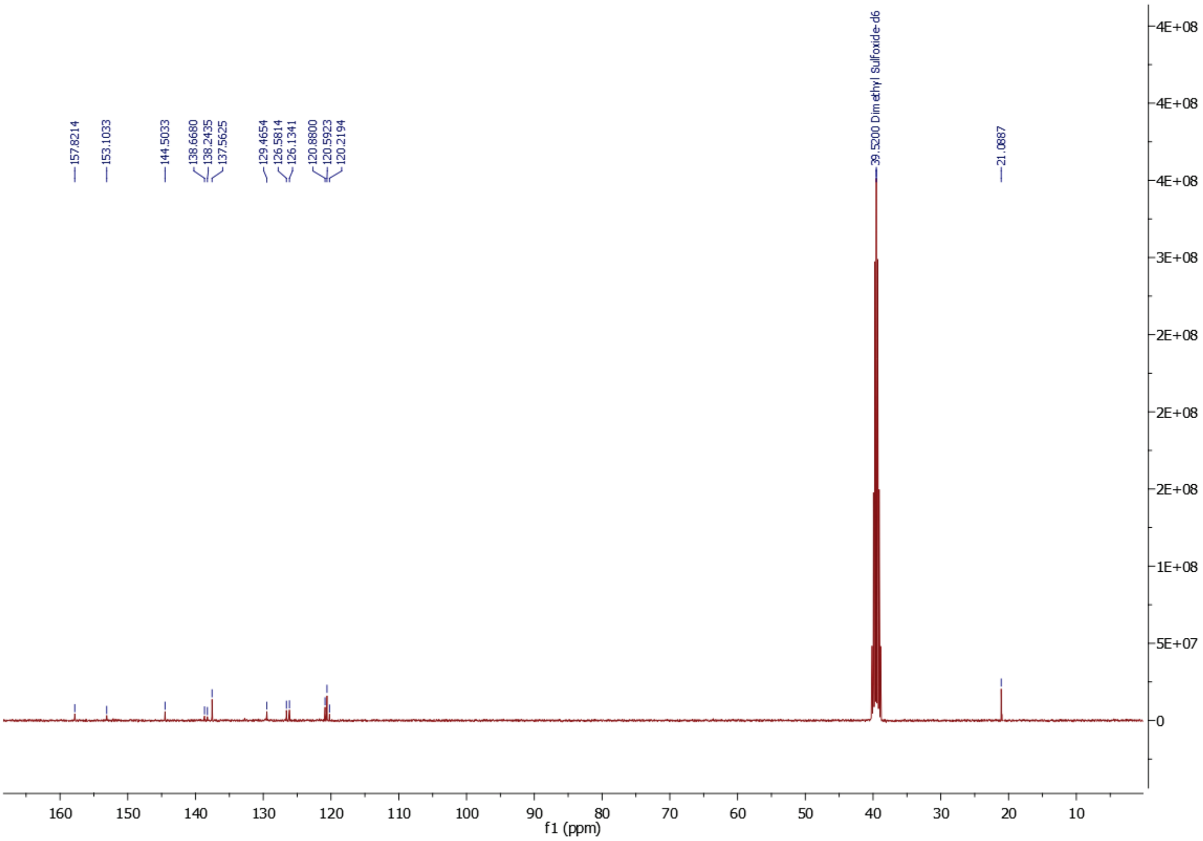

*N*[4]-(3,4-Dimethylphenyl)quinazoline-4,6-diamine **IIIf**

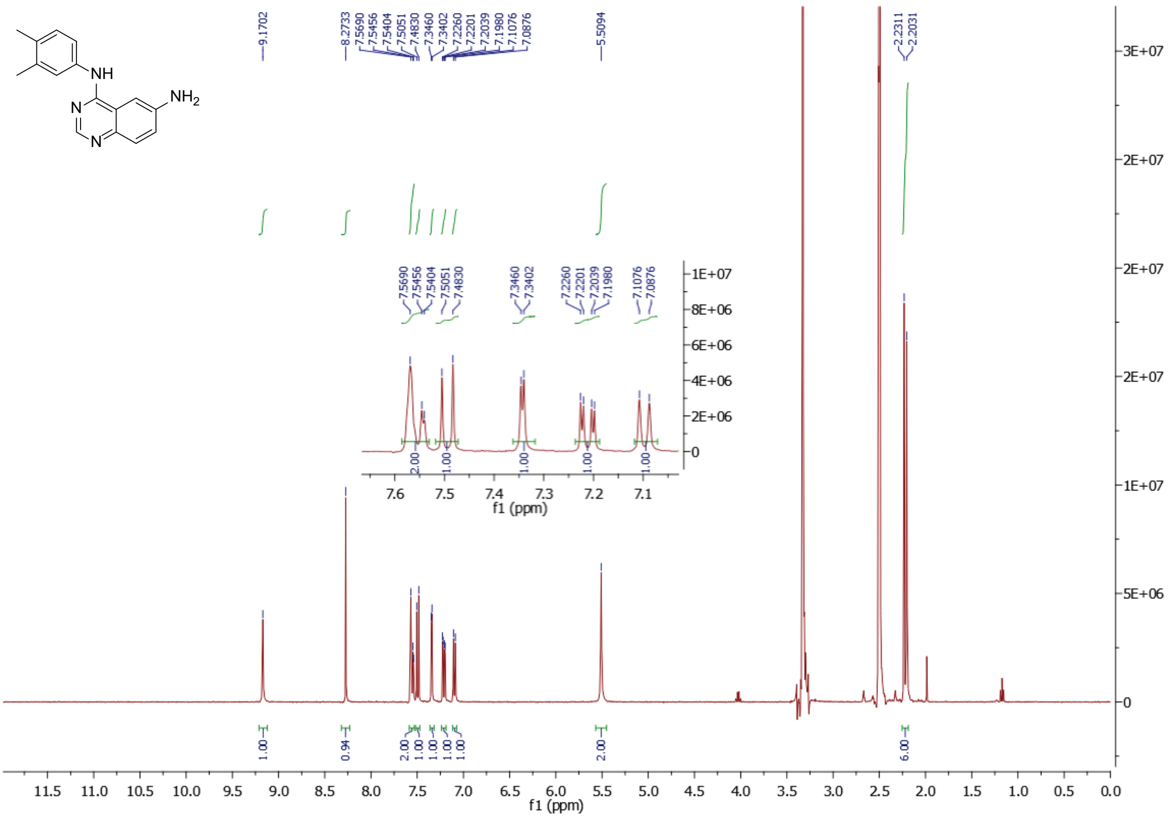


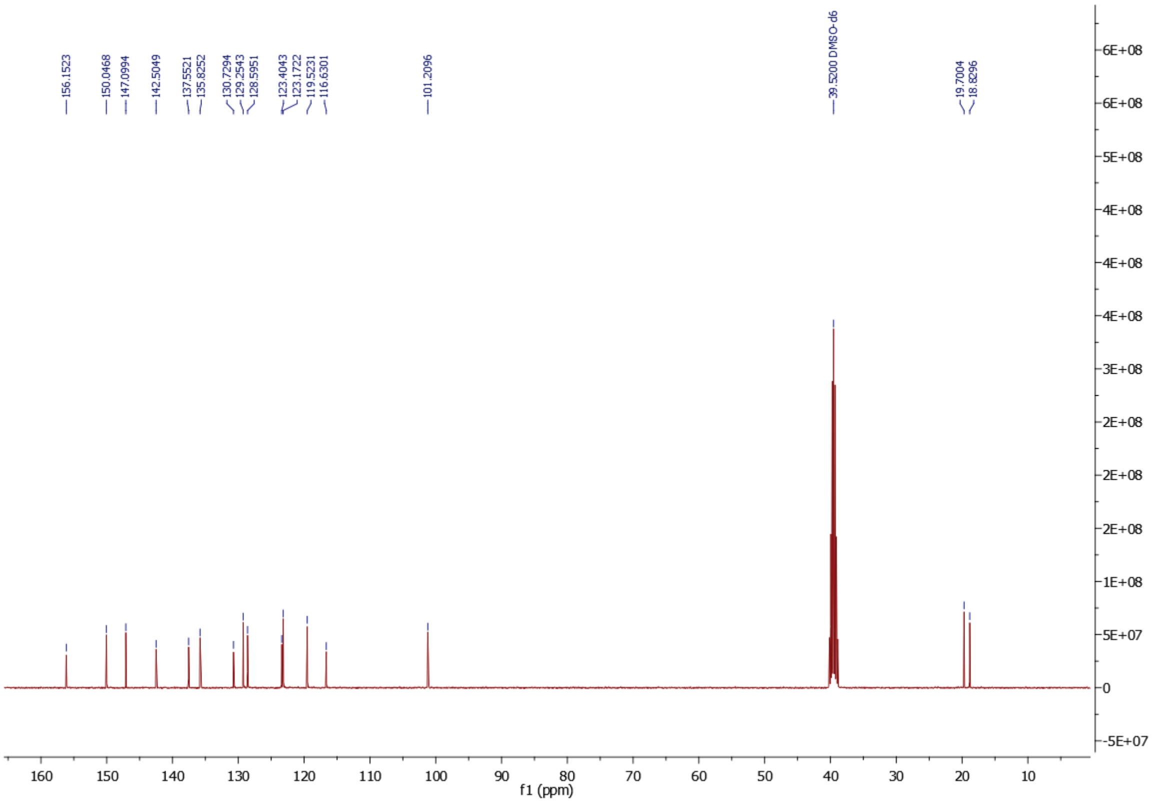

*N*[4]-(3,5-Dimethylphenyl)quinazoline-4,6-diamine **IIIg**

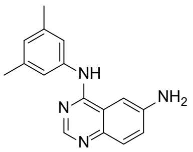


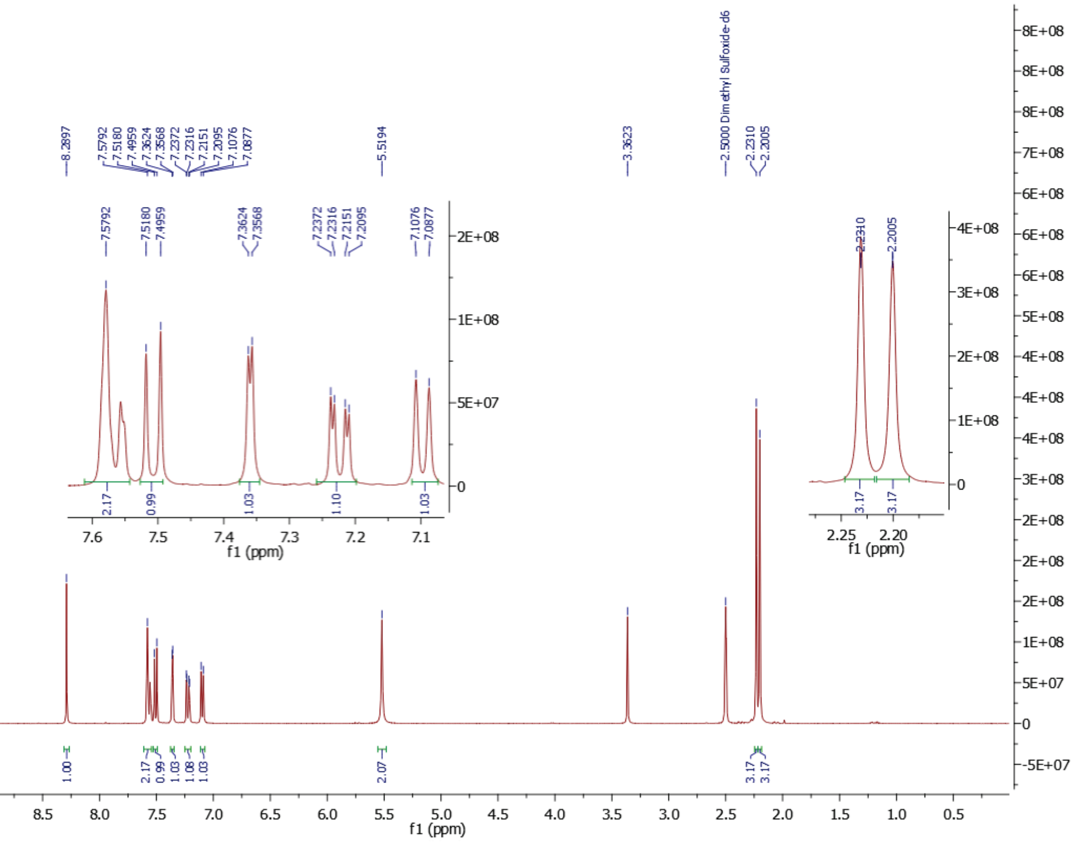


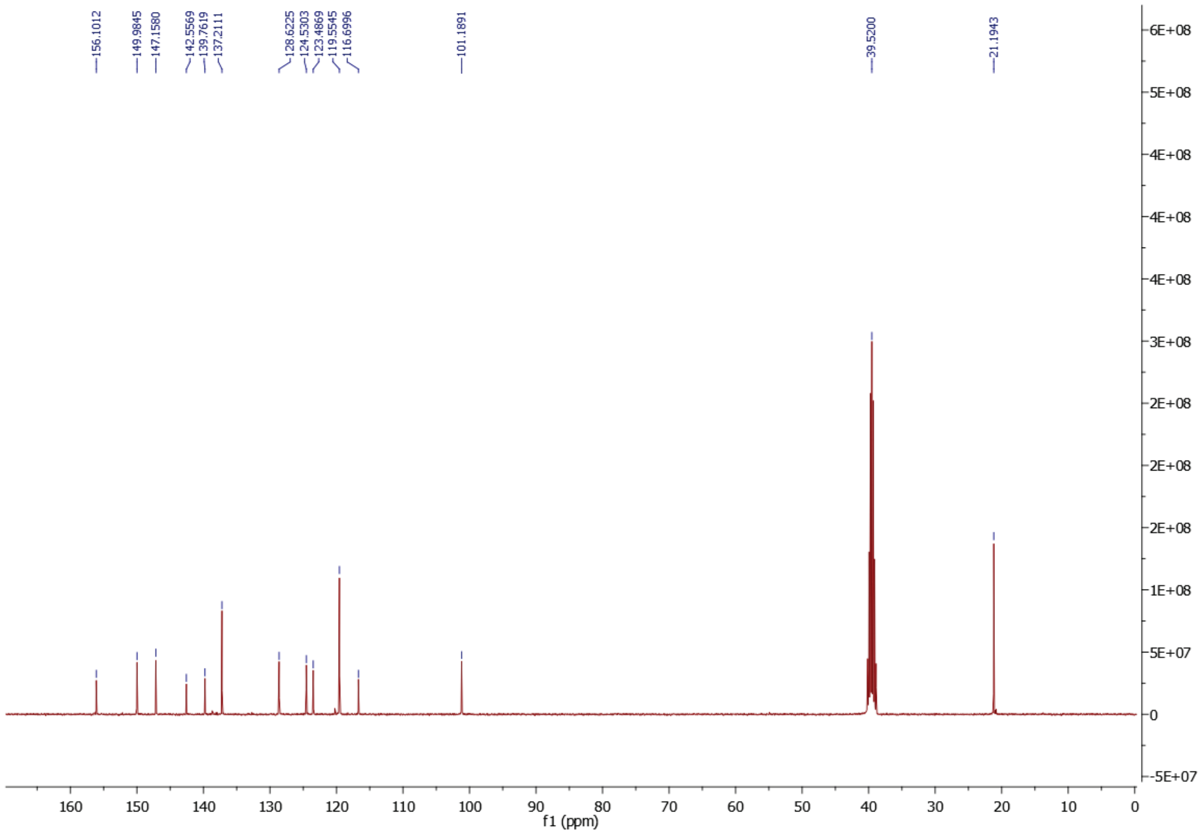

*N*[4]-(3,5-Dimethoxyphenyl)quinazoline-4,6-diamine ***IIIi***

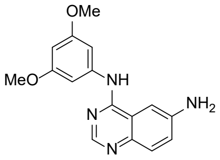


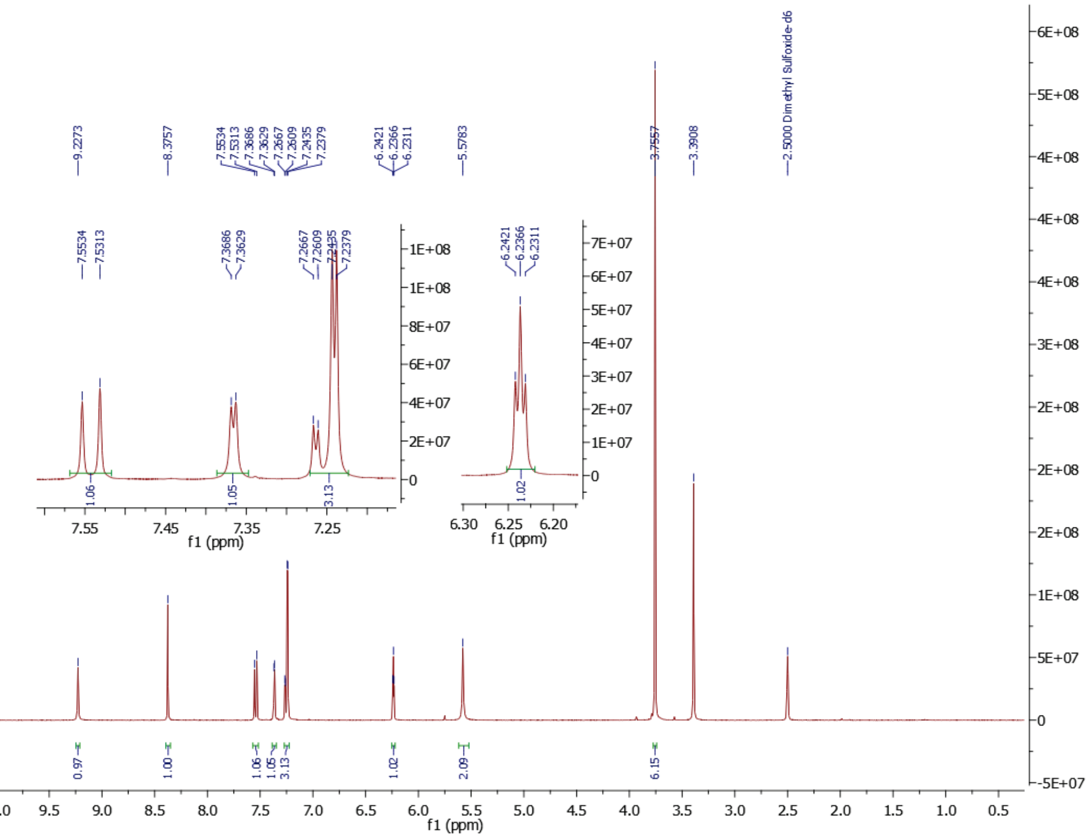


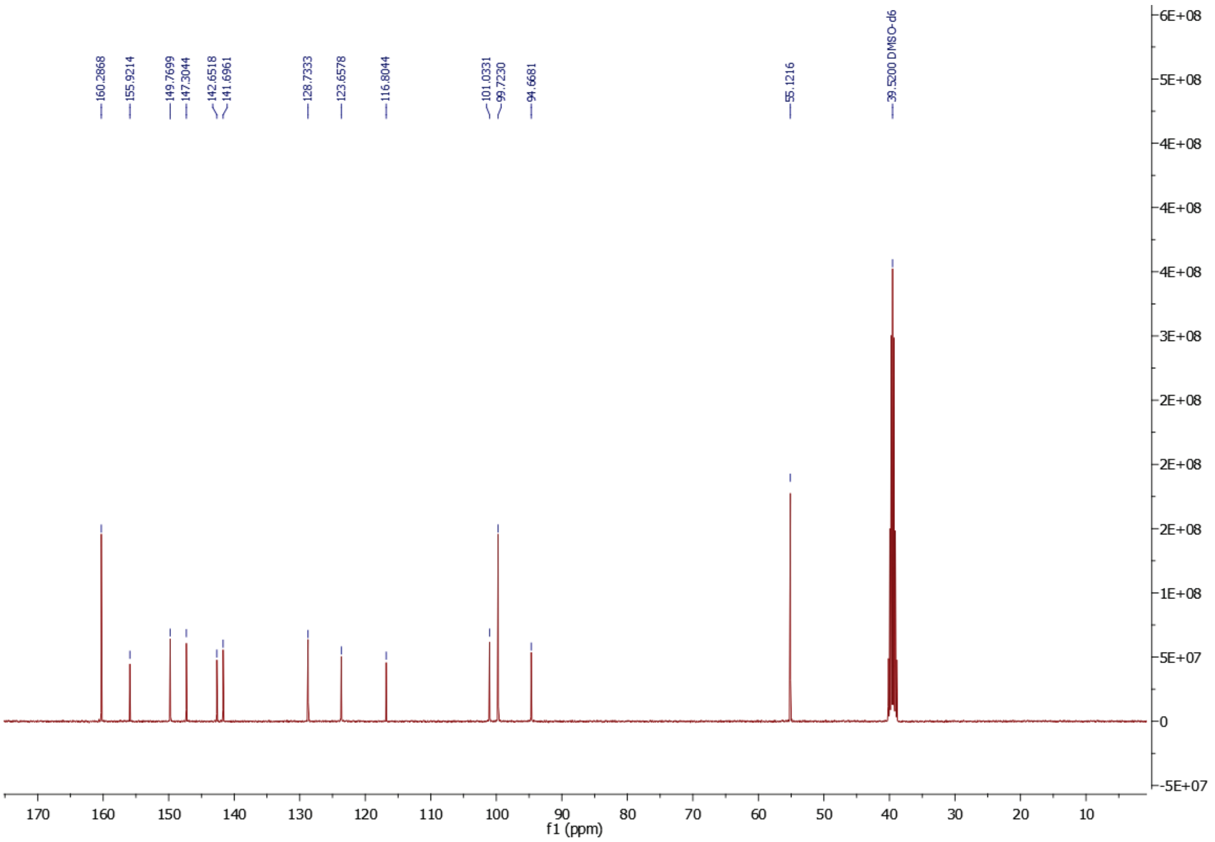

*(Z)-6-[(4-Chloro-5H-1,2,3-dithiazol-5-ylidene)amino]-N-phenylquinazolin-4-amine* ***5a***

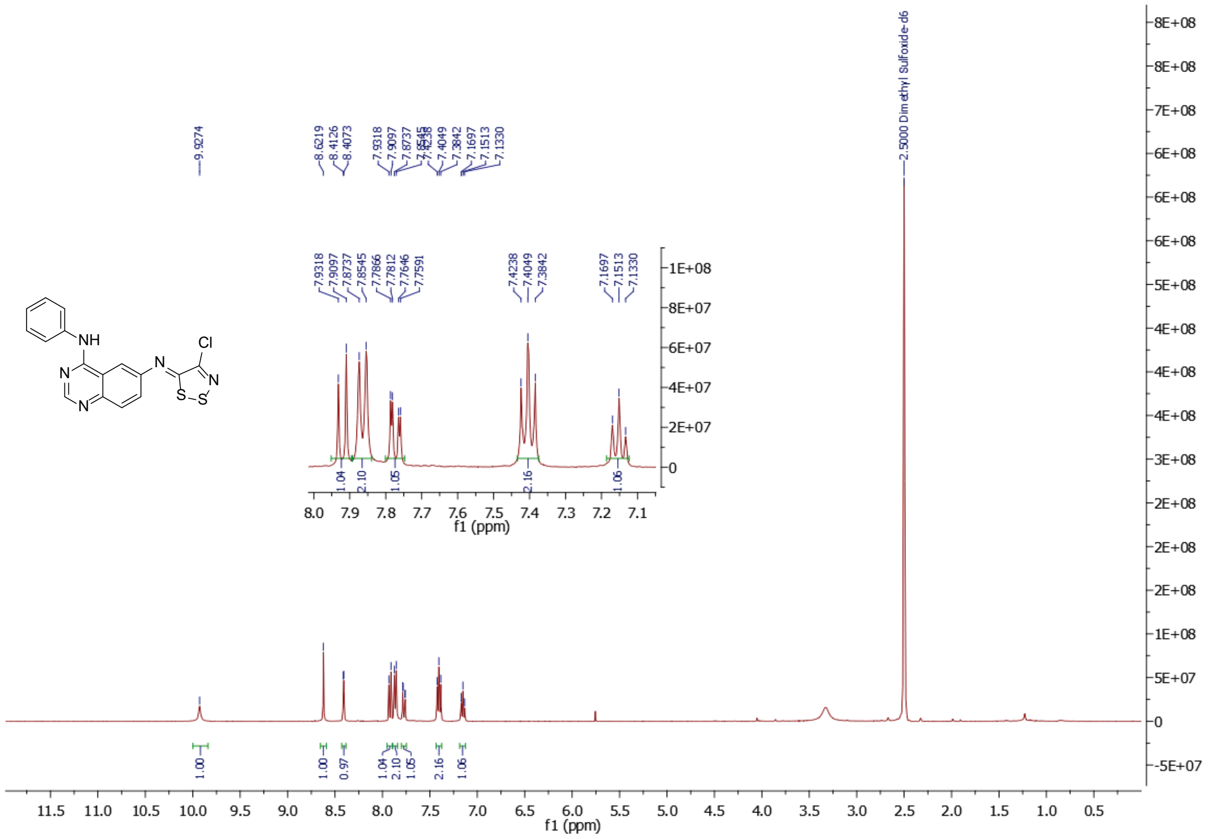


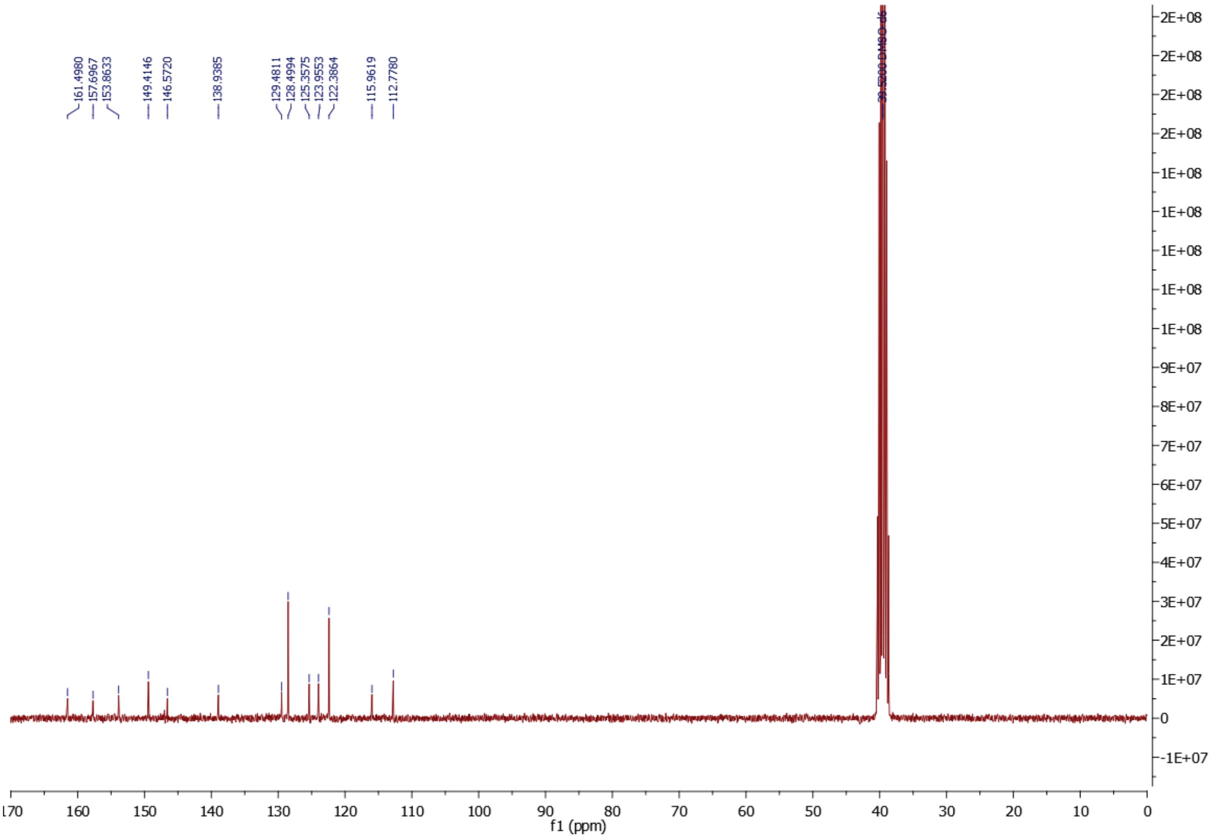

*(Z)-6-[(4-Chloro-5H-1,2,3-dithiazol-5-ylidene)amino]-N-(4-fluorophenyl)quinazolin-4-amine* ***5b***

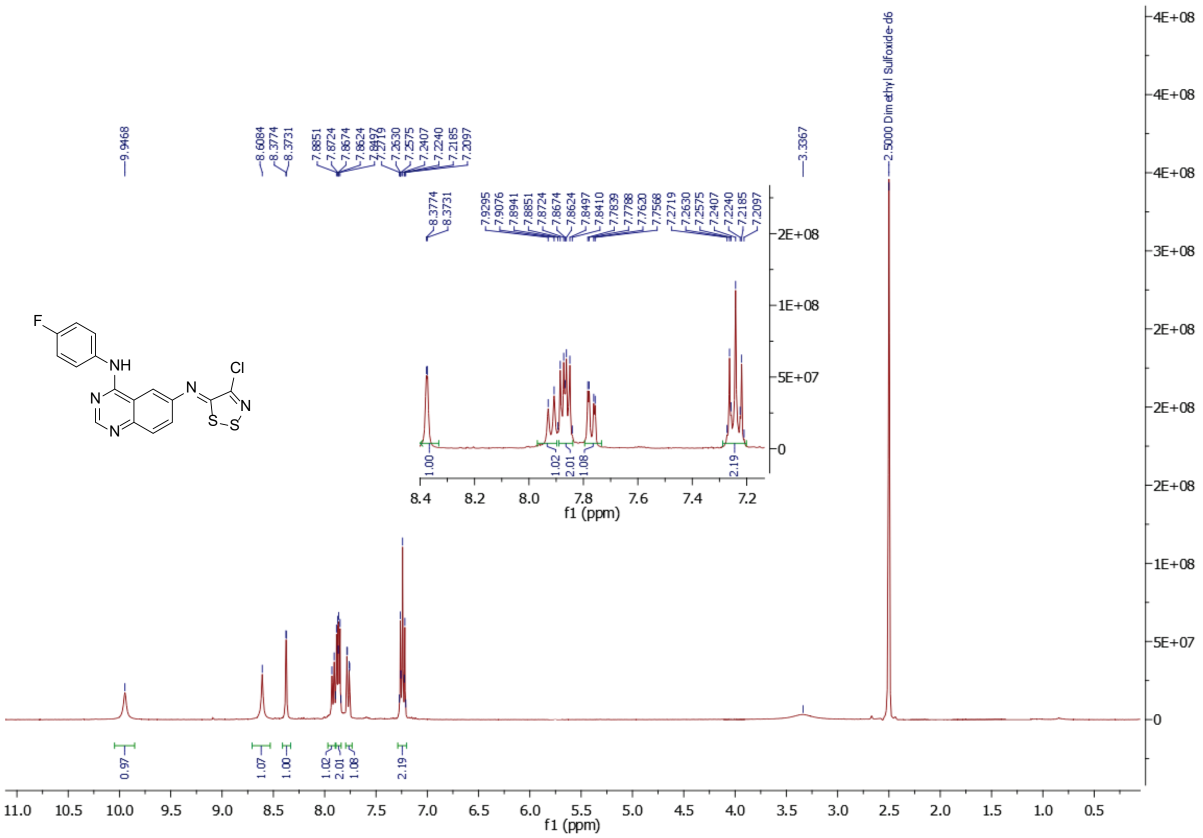


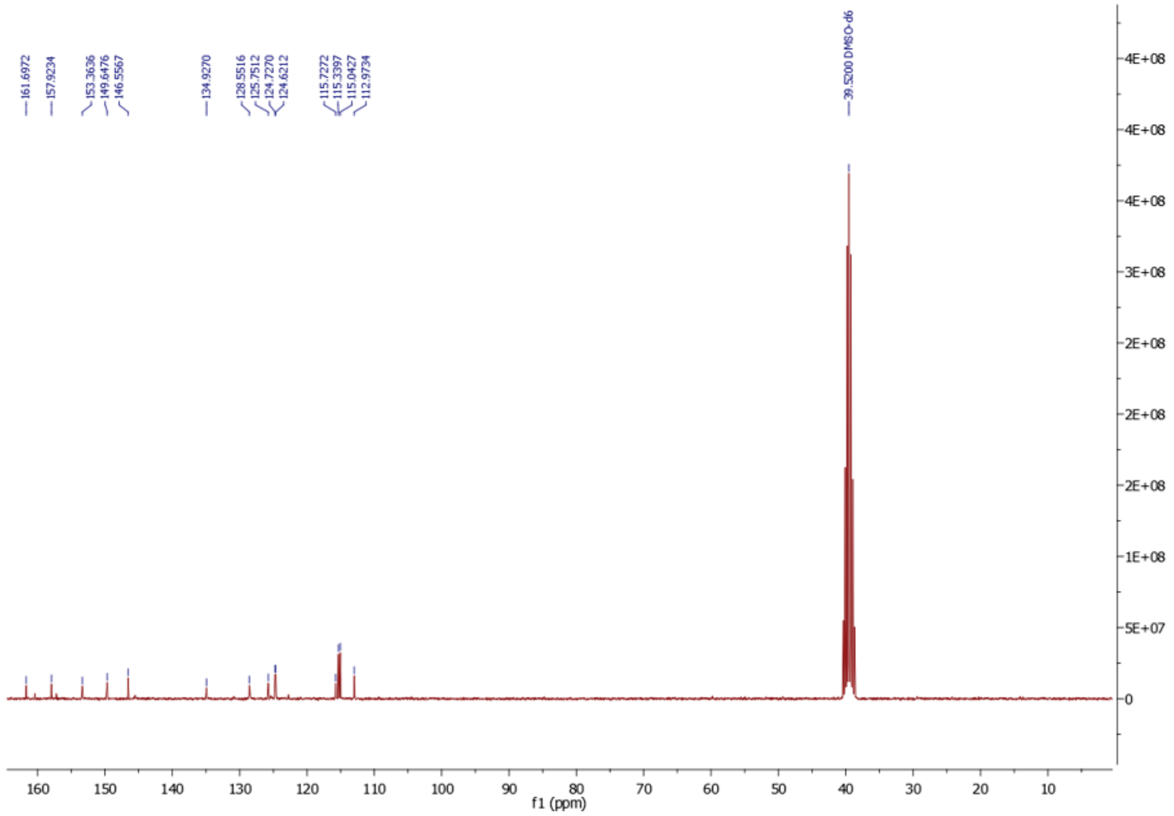

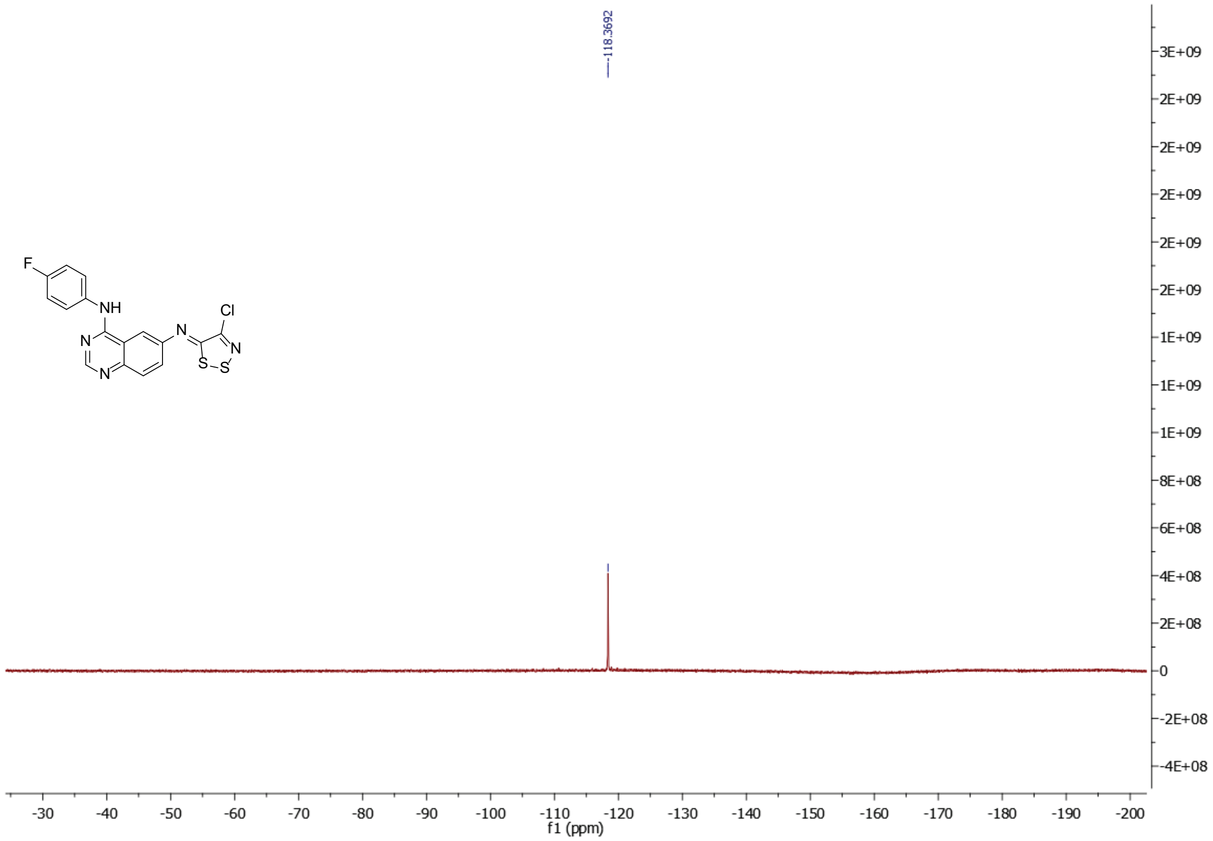
-118.3692
f1 (ppm)

*(Z)-6-[(4-Chloro-5H-1,2,3-dithiazol-5-ylidene)amino]-N-(p-tolyl)quinazolin-4-amine* ***5c***

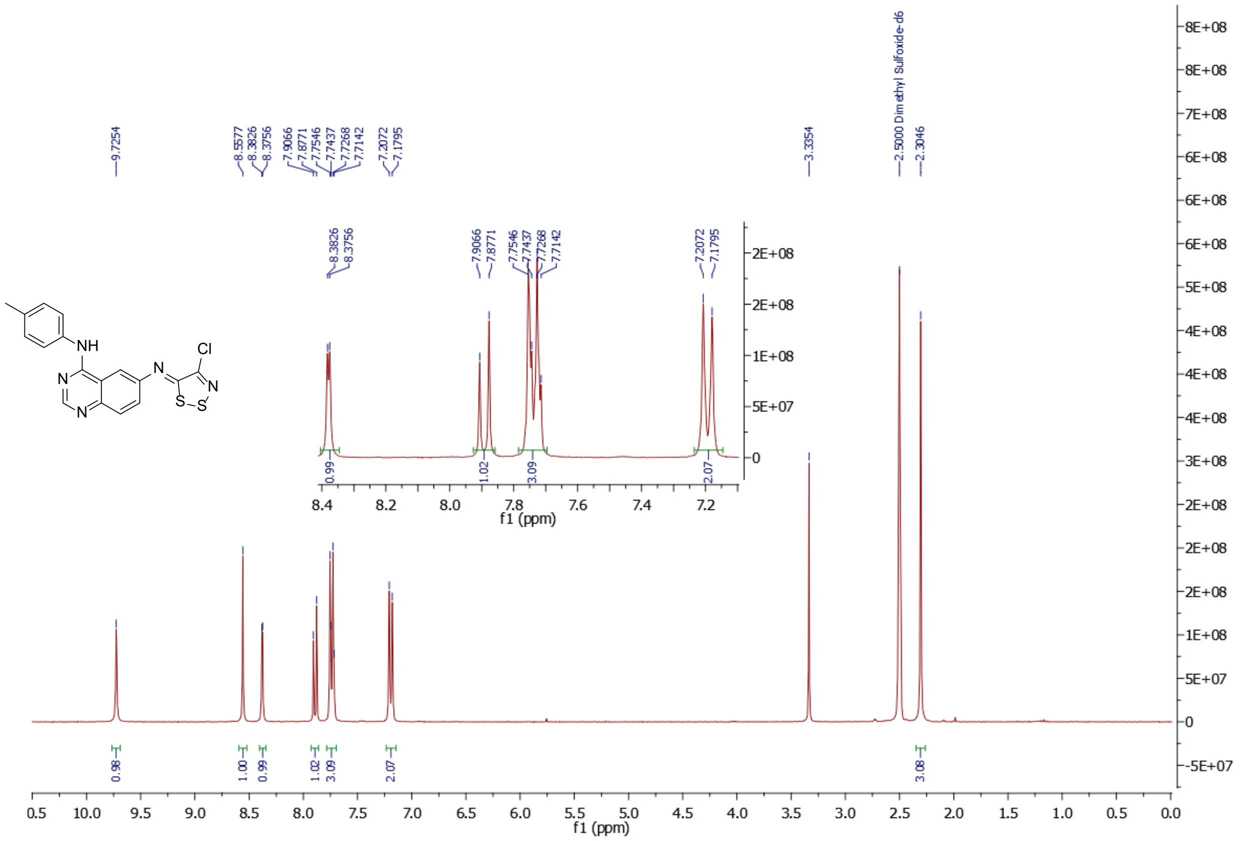


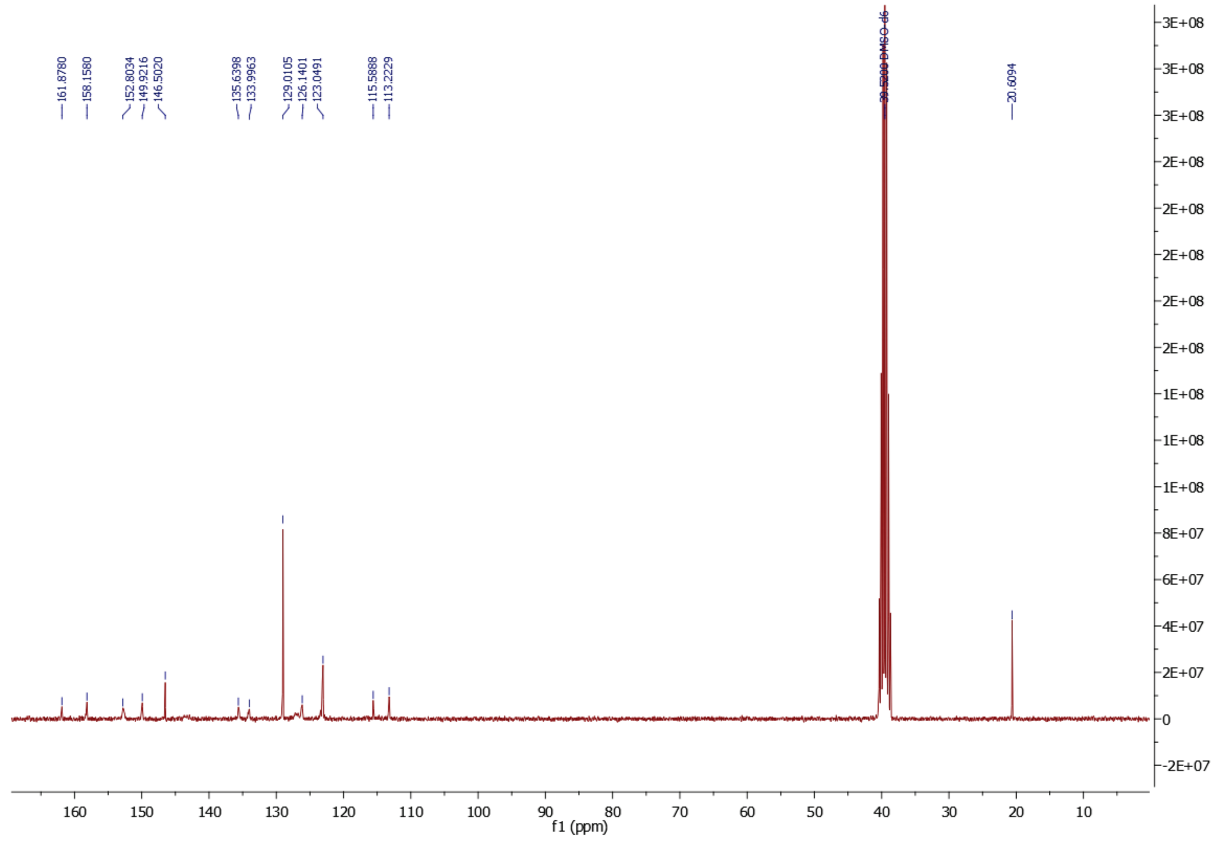

*(Z)-6-[(4-Chloro-5H-1,2,3-dithiazol-5-ylidene)amino]-N-(4-methoxyphenyl)quinazolin-4-amine* ***5d***

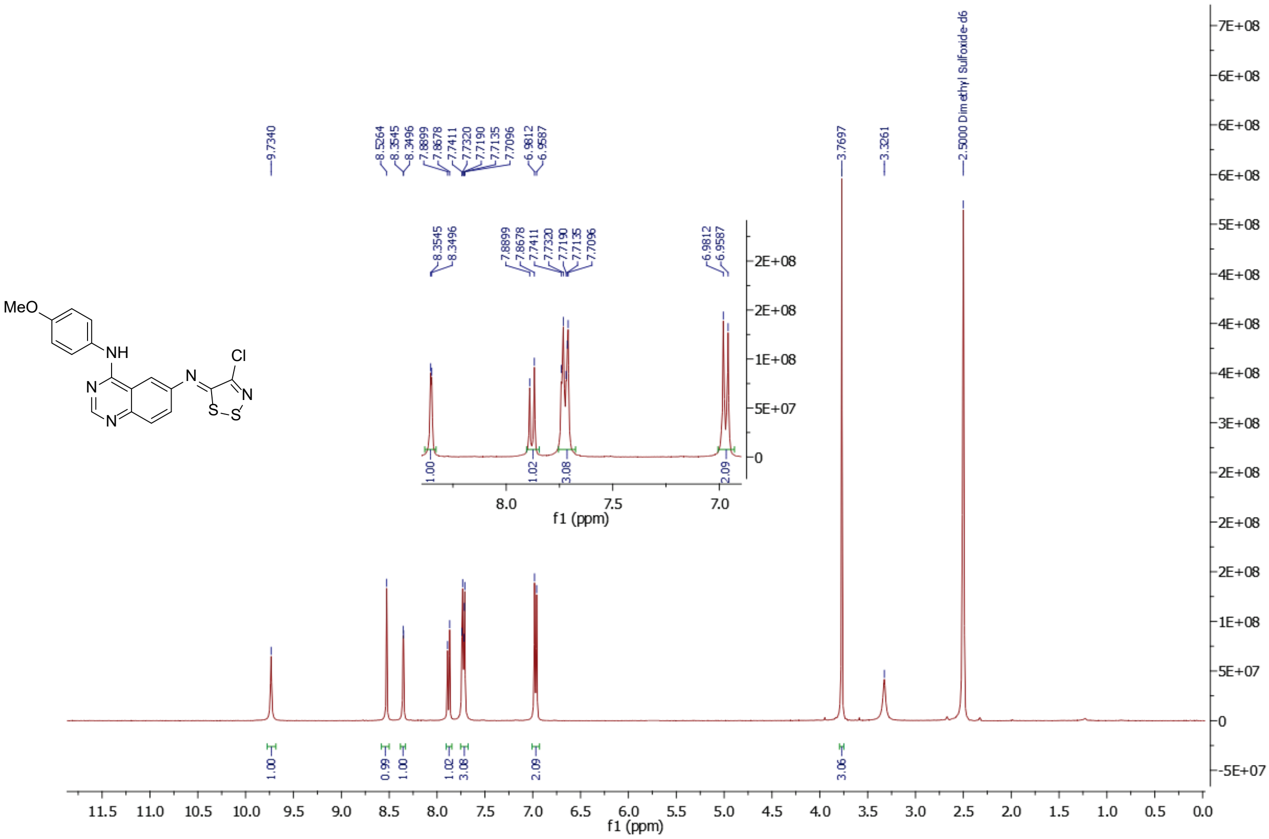


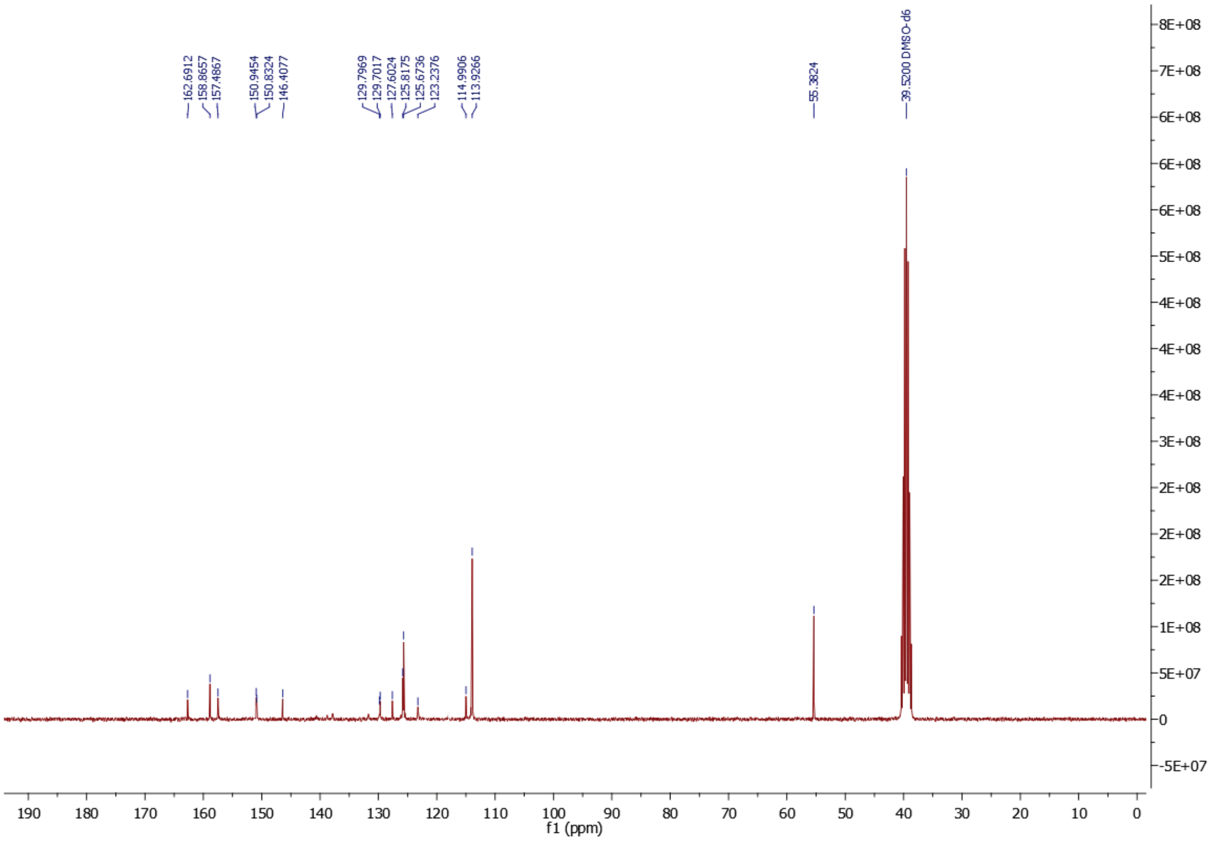

*(Z)-6-[(4-Chloro-5H-1,2,3-dithiazol-5-ylidene)amino]-N-(4-(trifuloromethyl)phenyl)quinazolin-4-amine* ***5e***

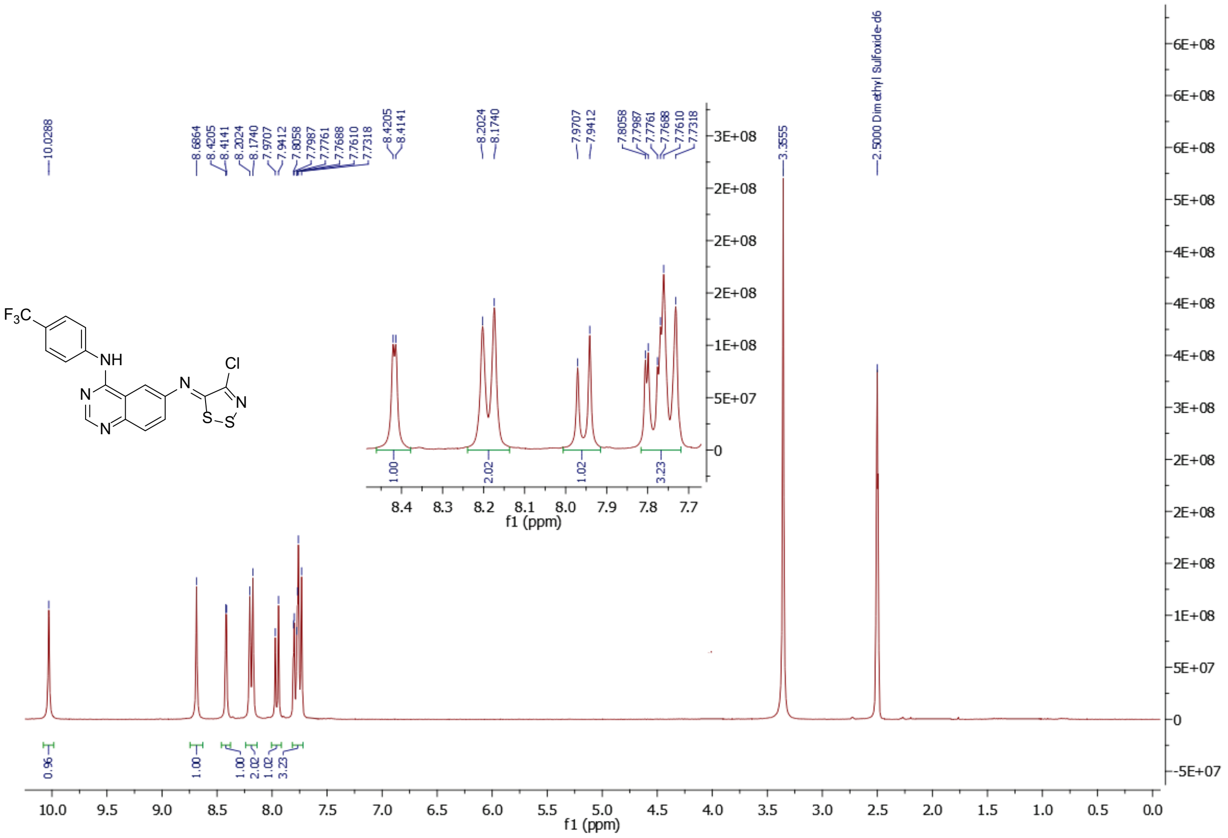


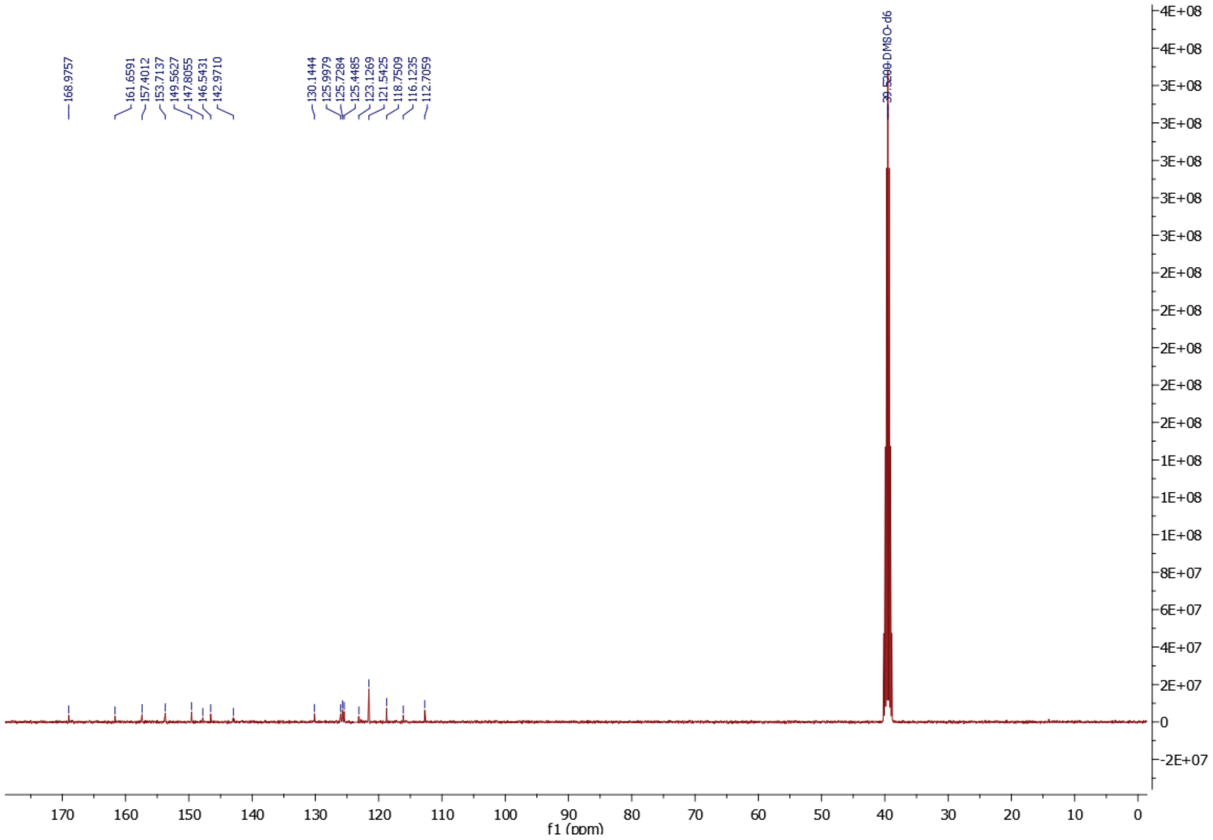

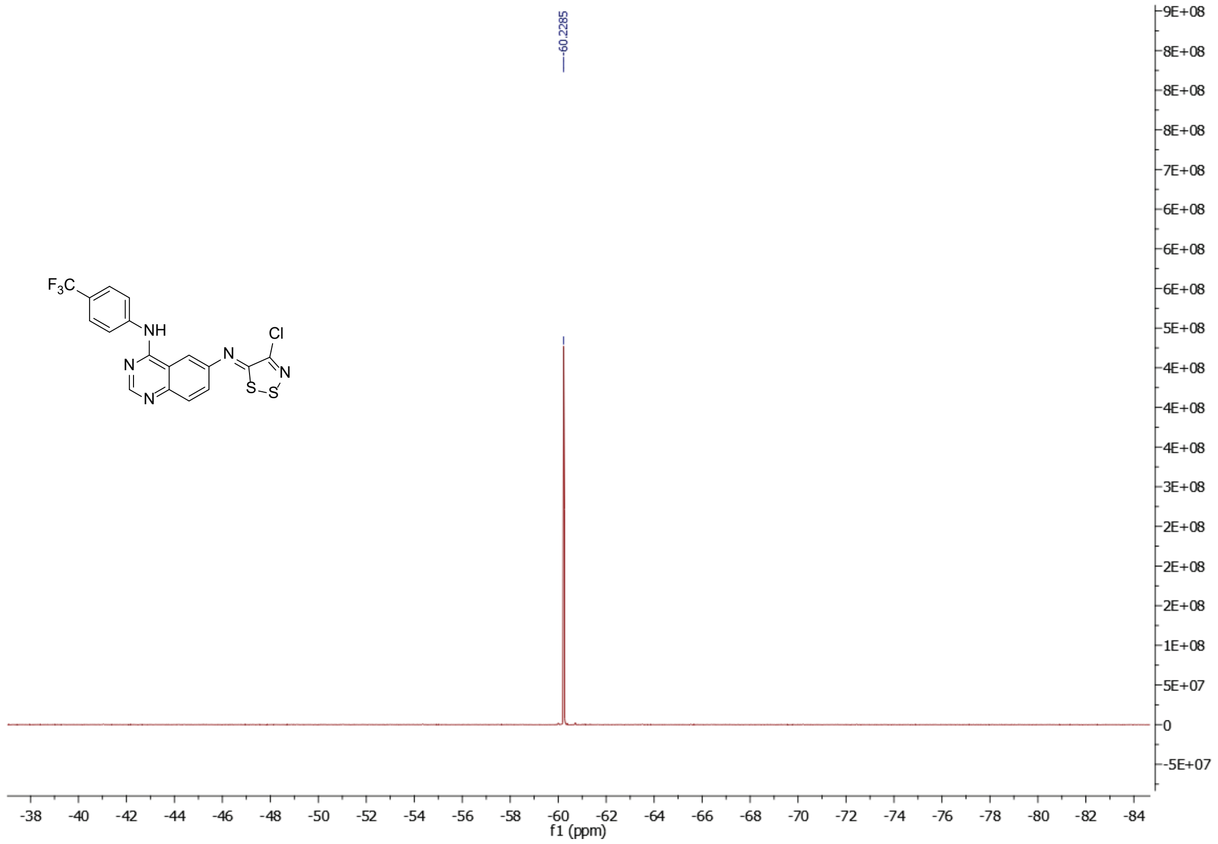

-60.2285
F3C
NH
N
N
N
Cl
N
S
S
9E+08
8E+08
8E+08
8E+08
7E+08
6E+08
6E+08
6E+08
5E+08
4E+08
4E+08
4E+08
3E+08
2E+08
2E+08
2E+08
1E+08
5E+07
0
-5E+07
-38 -40 -42 -44 -46 -48 -50 -52 -54 -56 -58 -60 -62 -64 -66 -68 -70 -72 -74 -76 -78 -80 -82 -84
f1 (ppm)

*(Z)-6-[(4-Chloro-5H-1,2,3-dithiazol-5-ylidene)amino]-N-(3,4-dimethylphenyl)quinazolin-4-amine* ***5f***

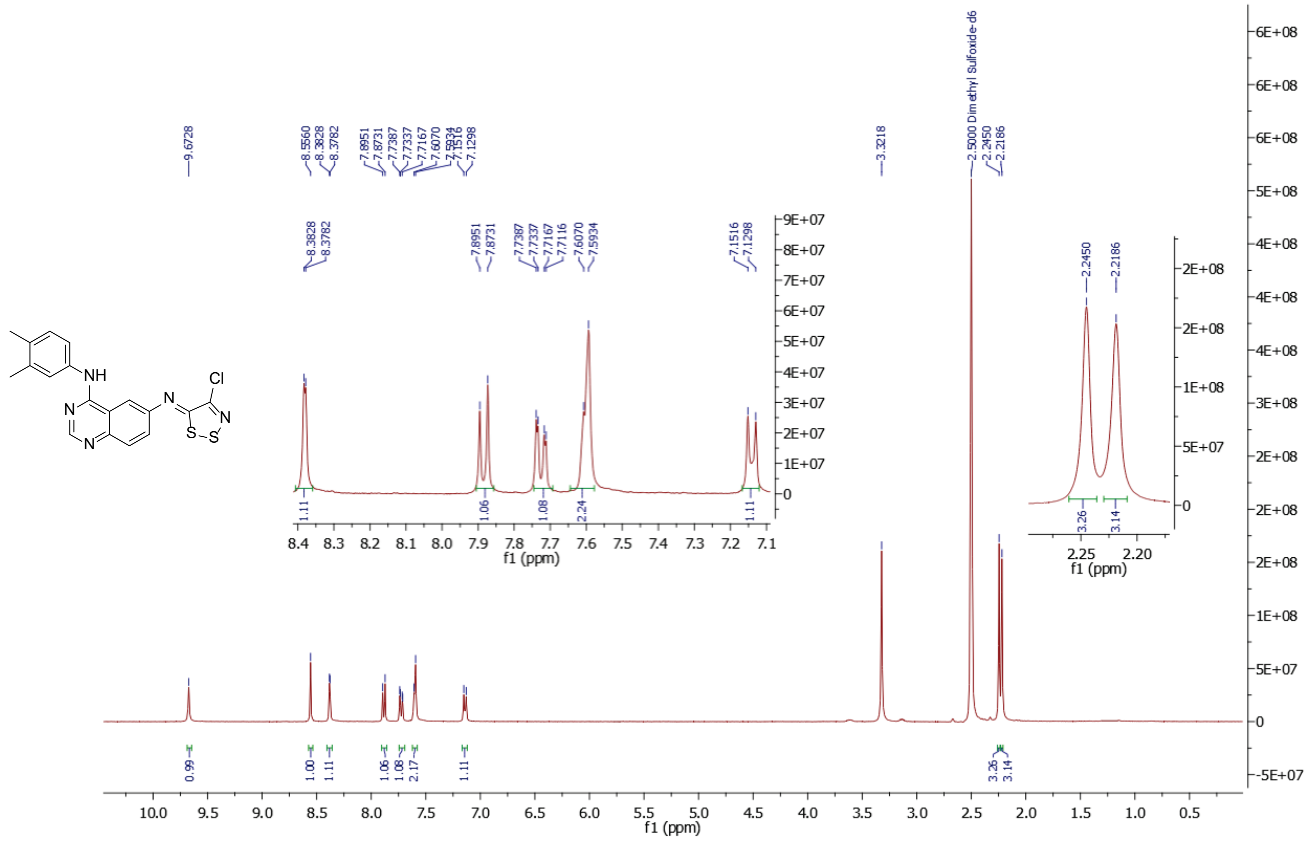

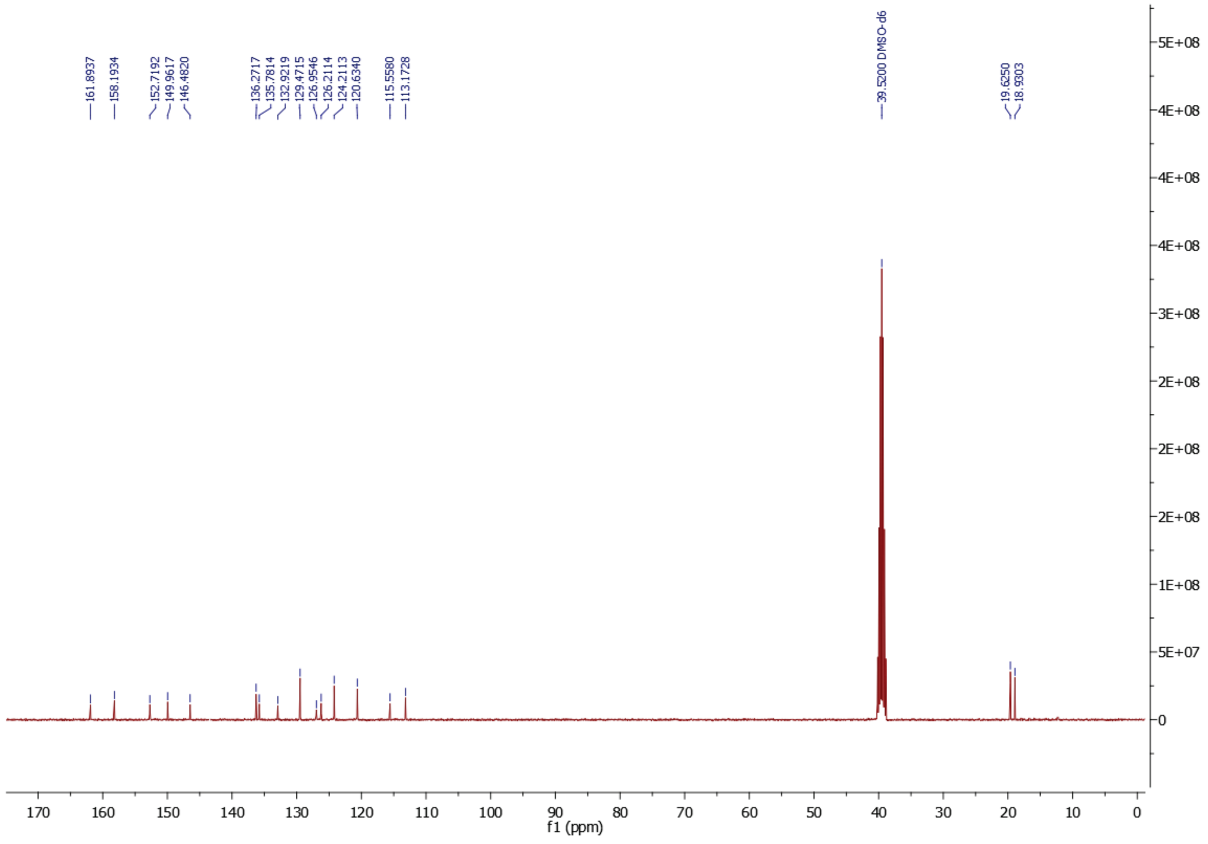

*(Z)-6-[(4-Chloro-5H-1,2,3-dithiazol-5-ylidene)amino]-N-(3,5-dimethylphenyl)quinazolin-4-amine* ***5g***

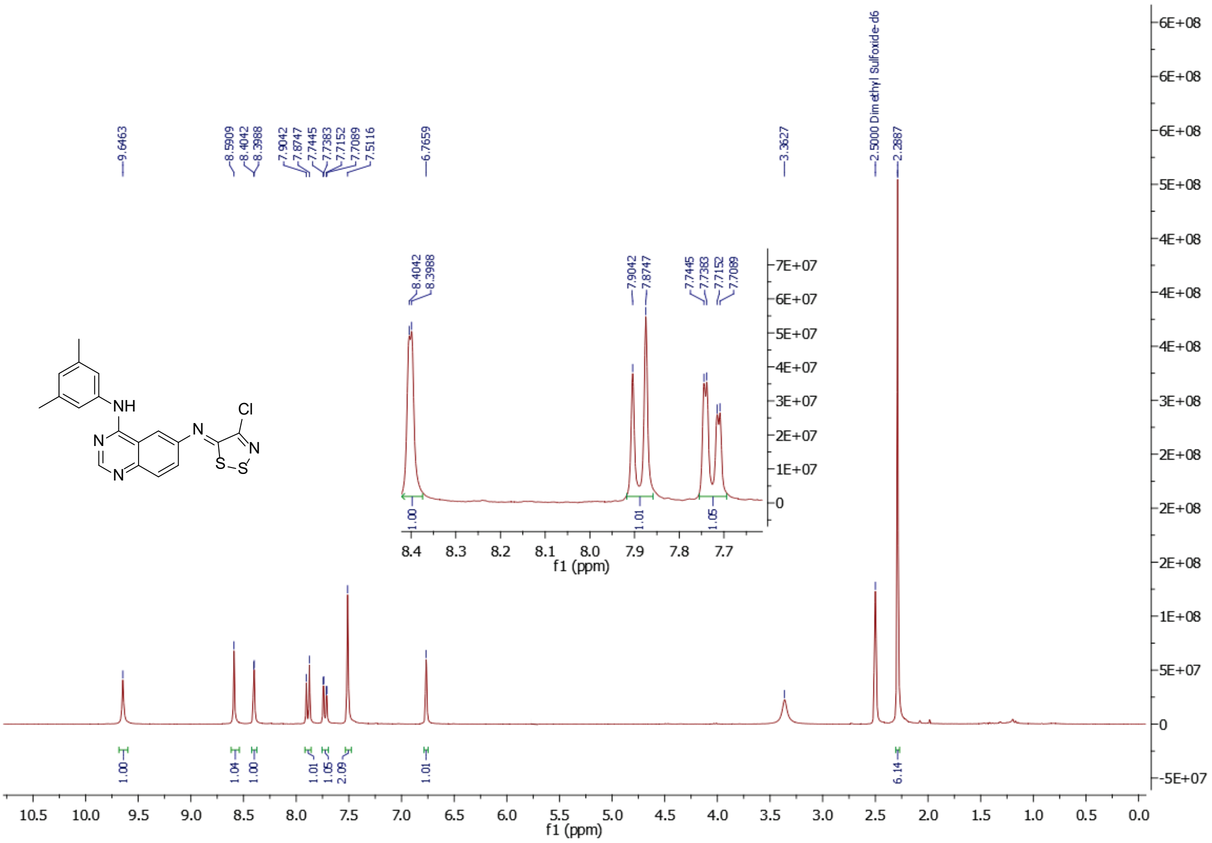


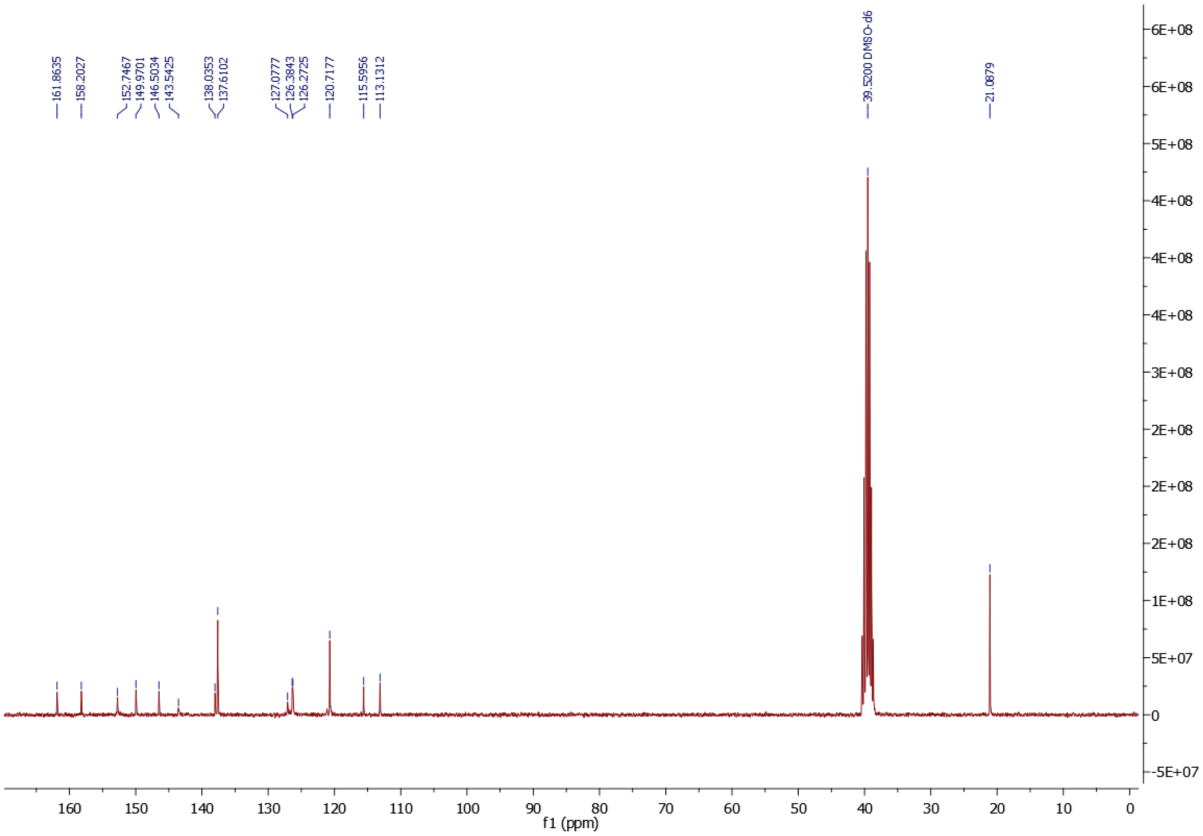

*(Z)-6-[(4-Chloro-5H-1,2,3-dithiazol-5-ylidene)amino]-N-(3,4-dimethoxyphenyl)quinazolin-4-amine* ***5h***

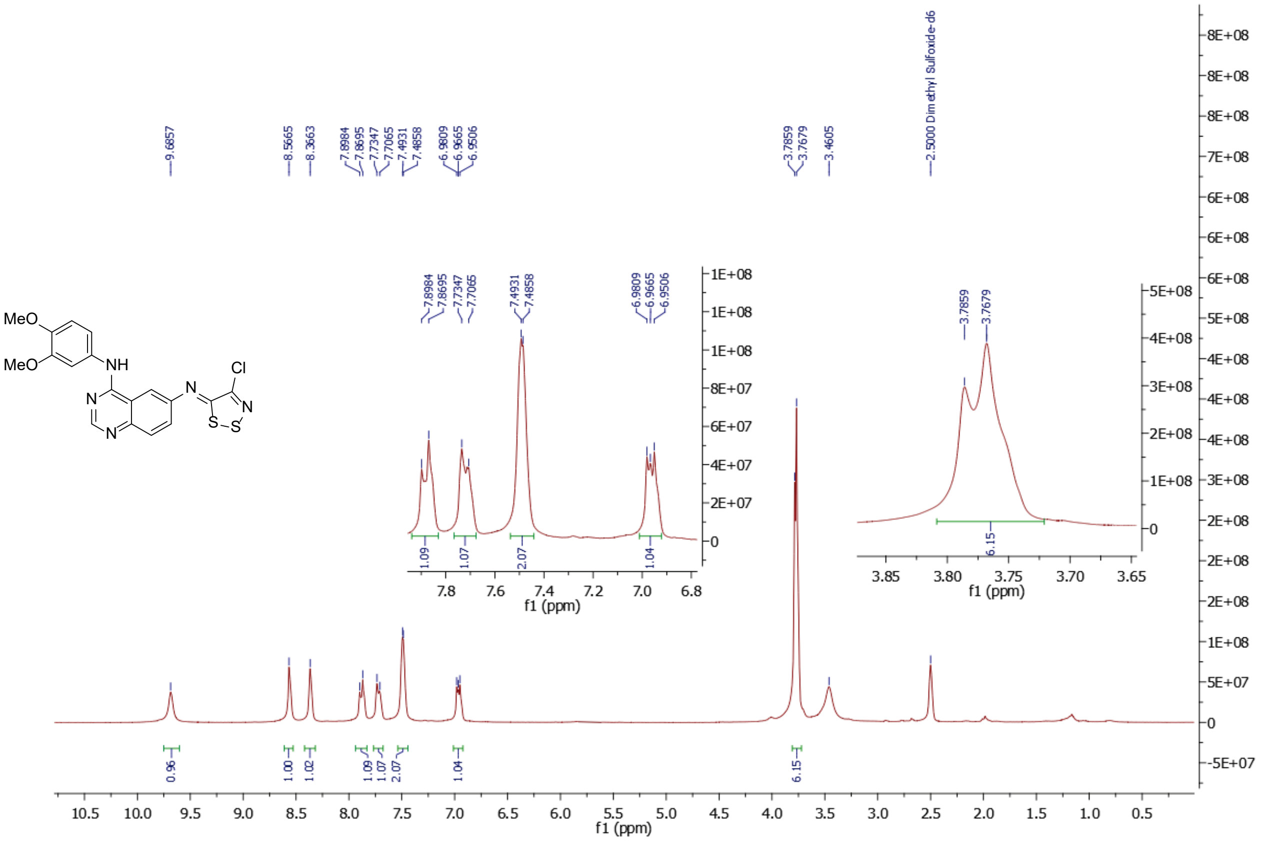


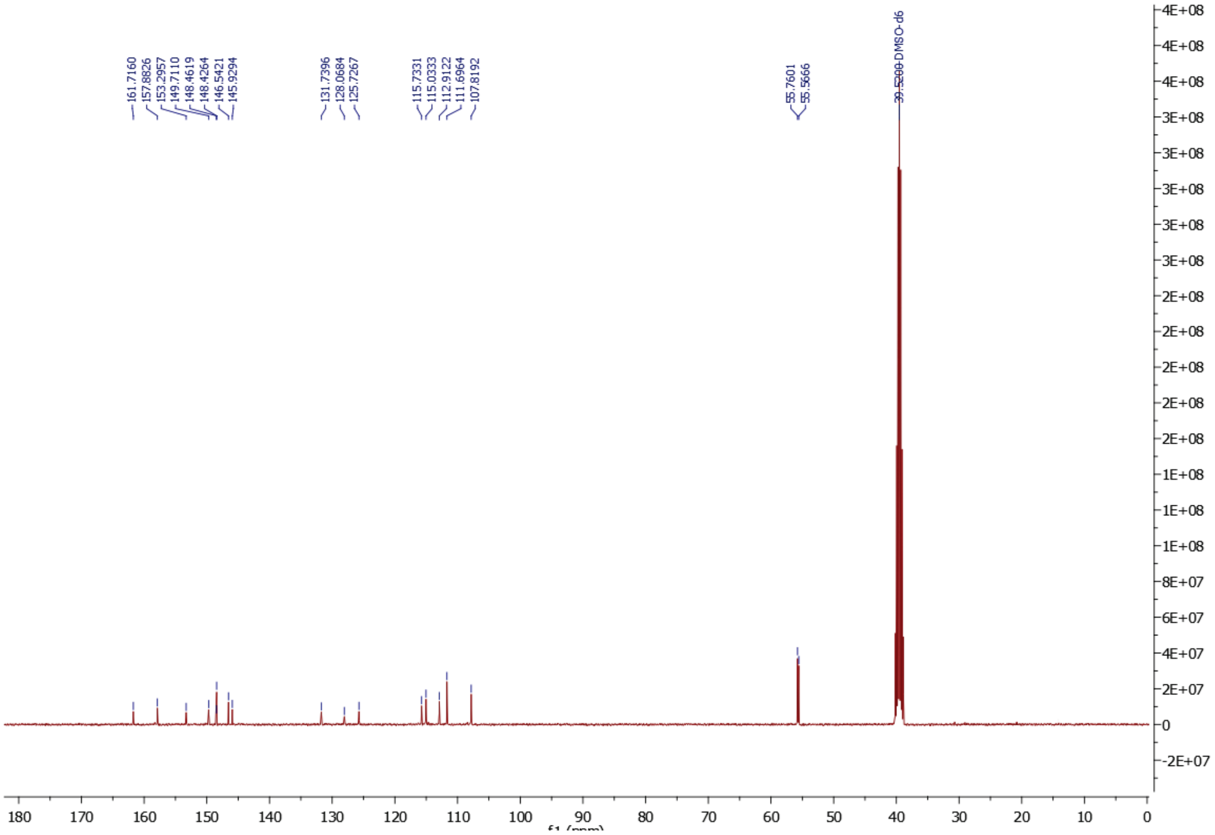

*(Z)-6-[(4-Chloro-5H-1,2,3-dithiazol-5-ylidene)amino]-N-(3,5-dimethoxyphenyl)quinazolin-4-amine* ***5i***

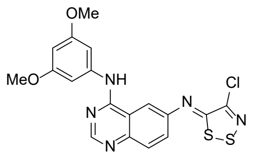


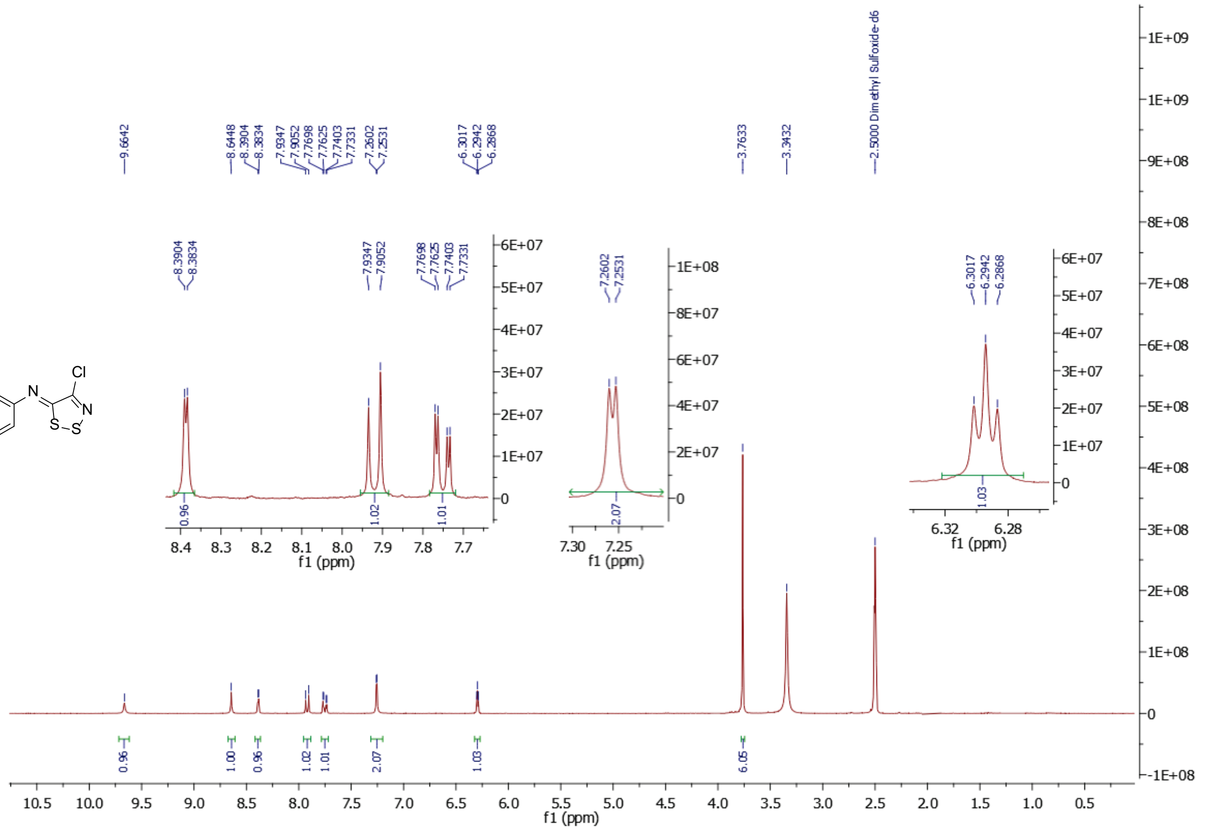

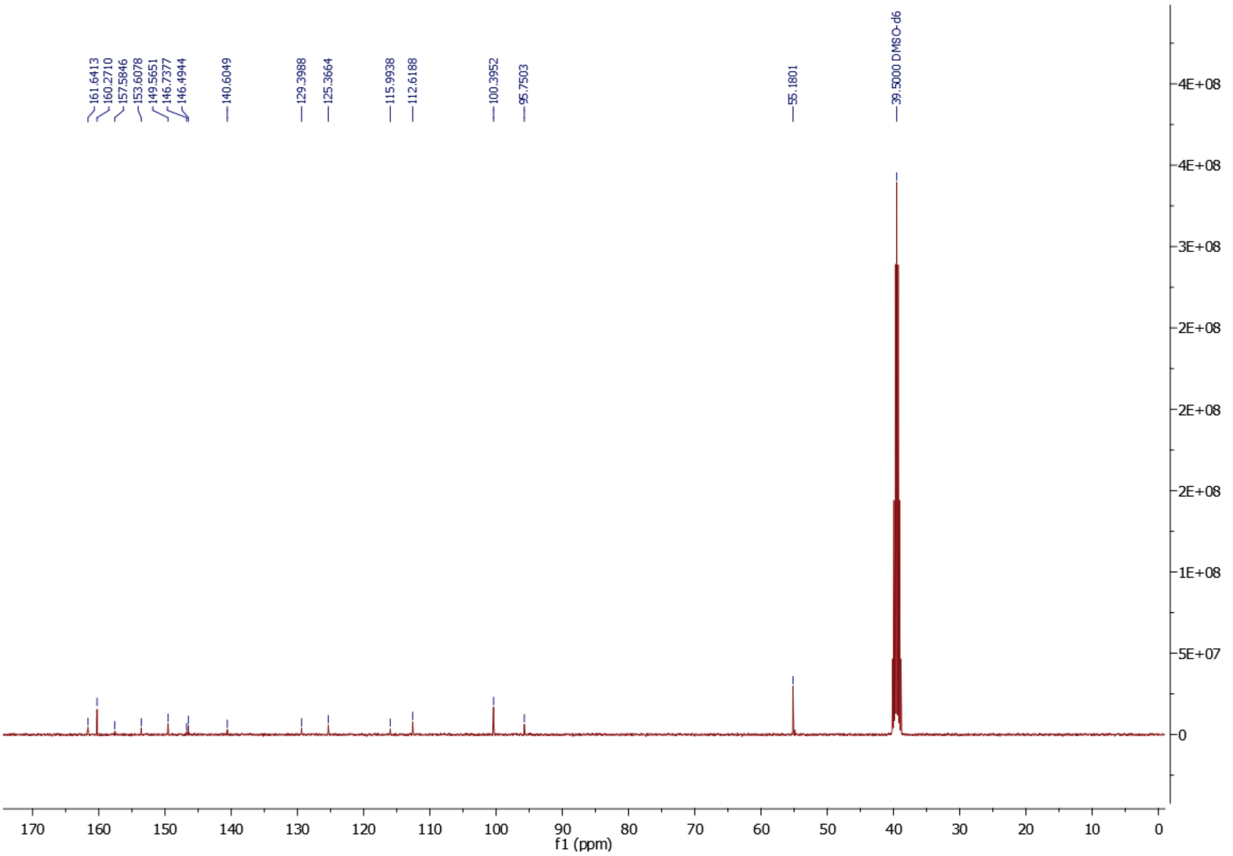

Supplement: Supplementary file 1 [file pharmaceuticals-18-01733-s001.zip › pharmaceuticals-3961462-supplementary.pdf]
